# The development of fluorescence turn-on probe for Al(III) sensing and live cell nucleus-nucleoli staining

**DOI:** 10.1038/srep34807

**Published:** 2016-10-10

**Authors:** Anoop Kumar Saini, Vinay Sharma, Pradeep Mathur, Mobin M. Shaikh

**Affiliations:** 1 Discipline of Chemistry, Indian Institute of Technology Indore, Simrol, Indore 453552, India; 2 Centre for Biosciences and Bio-Medical Engineering, Indian Institute of Technology Indore, Simrol, Indore 453552, India; 3 Metallurgical Engineering and Material Science, Indian Institute of Technology Indore, Simrol Indore 453552, India

## Abstract

The morphology of nucleus and nucleolus is powerful indicator of physiological and pathological conditions. The specific staining of nucleolus recently gained much attention due to the limited and expensive availability of the only existing stain “SYTO RNA-Select”. Here, a new multifunctional salen type ligand (**L**_**1**_) and its Al^3+^ complex (**1**) are designed and synthesized. **L**_**1**_ acts as a chemosensor for Al^3+^ whereas **1** demonstrates specific staining of nucleus as well as nucleoli. The binding of **1** with nucleic acid is probed by DNase and RNase digestion in stained cells. **1** shows an excellent photostability, which is a limitation for existing nucleus stains during long term observations. **1** is assumed to be a potential candidate as an alternative to expensive commercial dyes for nucleus and nucleoli staining.

Recent advancement and development in fluorescence imaging techniques has witnessed tremendous upsurge in the field of metal ion sensing and live cell imaging^1^,^2^,^3^,^4^,^5^. The sensitive bio-imaging of Al^3+^ in various cell lines can be helpful for understanding the fundamental mechanism of aluminium-induced human diseases. The widespread prevalence of Aluminium and its serious health threats such as Parkinson’s disease, Kidney damage and Alzheimer’s disease makes it imperative to develop Al(III) sensors^6^,^7^,^8^,^9^,^10^. Tolerable limit of Al^3+^ in human is estimated to be around 7 mg Kg^−1^ per week^11^. The conventional methods for Al^3+^ detection like inductively coupled plasma-atomic emission spectrometry (ICP-AES), atomic absorption spectrometry (AAS) and inductively coupled plasma-mass spectrometry(ICP-MS) are relatively expensive and suffers from lack of selectivity, sensitivity and interferences generated from the matrix^12^. However, spectrofluorimetry technique is superior in terms of quick analysis, high selectivity, sensitivity and ease of operation^13^. On the other hand, the critical role of cell nucleus in various cellular events like metabolism, reproduction and heredity highlights the importance of nucleus staining agents for determining the morphology and functionality of nucleus^14^. The existing nuclear staining agents like DAPI and Hoechst suffers from photo-bleaching and self-quenching which limits their applicability for long term observations^15^,^16^. The nucleolus is an important sub-nuclear structure and its dynamic morphology is indicative of pathological and physiological situations^17^. The inability of DAPI and Hoechst to stain nucleolus and limitations of extremely expensive existing nucleolus stains emphasize the requirement of a total nucleus staining dye. Hence, the development of new generation of low cytotoxic and high photostable nucleus staining agent^16^ as well as selective and sensitive sensor for intracellular detection of trace amount of Al^3+^ accorded significant attention^18^,^19^,^20^.

There are few reports on specific staining of nucleus by employing nano-materials^21^ and Ir(III)^14^/ Ru(II)^15^ complexes to different cell lines. Specific staining of nucleolus is also achieved using carbon based nano-materials^22^, nanoclusters^23^, coumarin and pyronin based moieties^24^,^25^, Eu(III)^26^/Ir(III)^27^ complexes and semiconducting quantum dots^28^. Two water soluble molecules containing N-methyl benzothiazolium moiety are recently reported which individually stains nucleus and nucleolus^29^. There exists only few commercial dye(s) which specifically stains nucleoli but has some limitations like specific storage requirements, toxicity, rare availability and expensiveness^14^. However, to the best of our knowledge, so far no report is available on specific nucleus as well as nucleoli staining using **1**.

Herein, we report synthesis of multifunctional ligand **L**_**1**_ and its Al^3+^ complex (**1**) for specific nucleus and nucleoli staining along with sensitive intracellular Al^3+^ detection.

## Results

**Figure 1:**
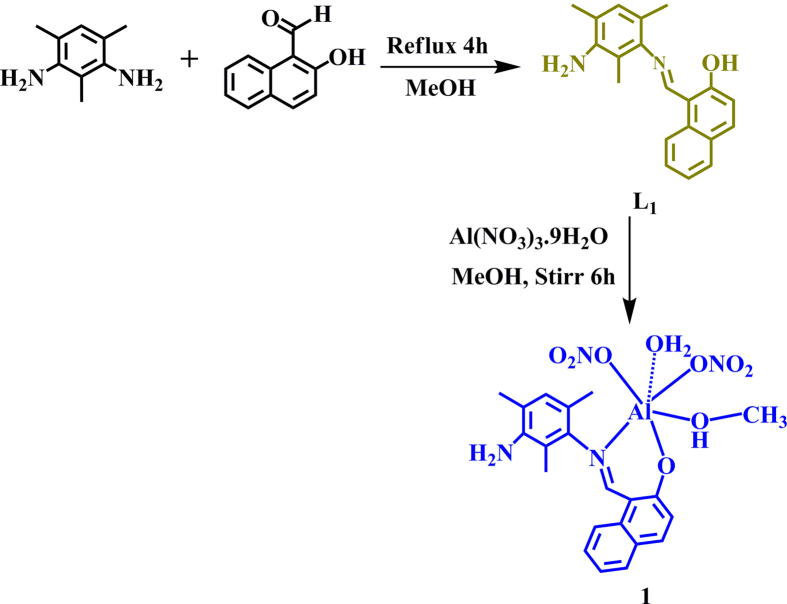
Synthesis of ligand L_1_ and 1.

We recently reported synthesis of a highly selective, sensitive and reversible symmetric chemosensor H_2_L for Zn^2+^ ion (Supplementary Fig. S1)^30^. Encouraged by the results, we have slightly altered the ratio of 2,4,6-trimethylbenzene-1,3-diamine and 2-Hydroxy-1-napyhaldehyde to 1:1 (Fig. 1) instead of 1:2 ratio, which results in another chemosensor (**L_1_**) for selective and sensitive detection of Al^3+^ which results in another chemosensor (**L_1_**) for selective and sensitive detection of Al^3+^.

**L_1_** is characterized by elemental analysis, HRMS, NMR and further authenticated by single crystal X-ray studies. Furthermore, the reaction of **L_1_** with Al(NO_3_)3.9H_2_O in presence of methanol at room temperature yields complex **1** (Fig. 1). **L_1_** crystallize in monoclinic *P2_1_*/*n* space group (Supplementary Fig. S2 and Supplementary Table S1). The packing of **L_1_** reveals intermolecular H-bonding interaction and C-H···π interaction^31^ forming hydrogen bonded 2D-network (Supplementary Fig. S3 and Supplementary Table S2).

### Photophysical properties

**Figure 2:**
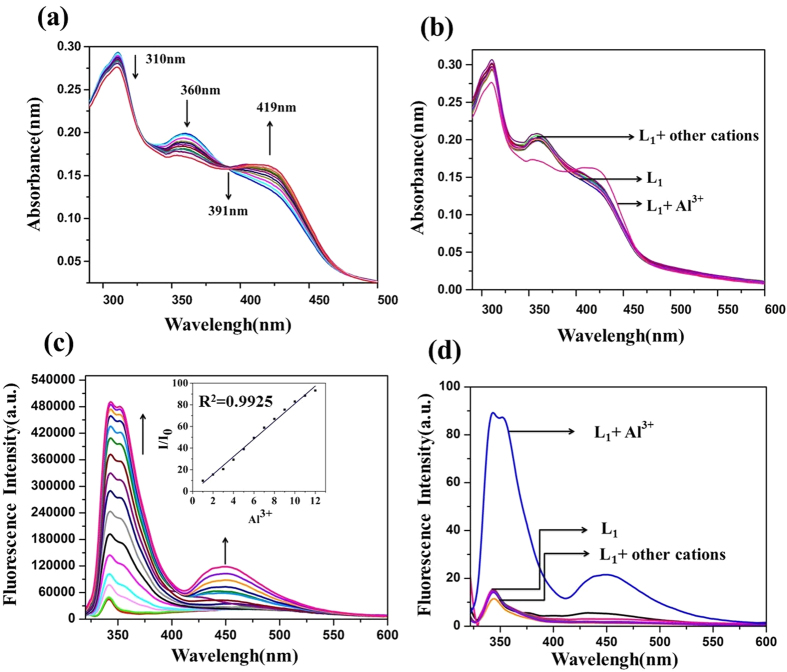
**(a)** UV-vis absorption titration spectra of L_1_ v/s Al^3+^. **(b)** Competitive absorption spectra of **L**_**1**_ in presence of different metal ions, **(c)** Fluorescence emission spectra obtained by the titration of **L**_**1**_ inset: shows the relative fluorescence intensity (I/I_0_) as a function of [Al^3+^]/[**L**_**1**_] mole ratio and **(d)** Effect of fluorescence intensity at 343 nm with addition of Al^3+^ along with other metal ions. (Conditions: (**L**_**1**_, c = 1.0** × **10^−5 ^M) in aq. ACN (ACN/H_2_O = 7:3 v/v, 10  μM HEPES buffer, pH = 7.4) with Al^3+^ ion(c = 1.0** × **10^−4^ M).

The absorption spectra of **L**_**1**_ shows absorption peaks at 309 nm and 360 nm corresponding to n-π* and at 419 nm corresponding to π-π* transition. Upon subsequent addition of increasing concentration of Al^3+^ ions, the absorbance intensity gradually enhanced at 419 nm and decreased at 309 and 360 nm having one isosbestic point at 391 nm (Fig. 2(a)) with change in color from *yellow* to *blue* under UV light. However, addition of varying concentrations of other metal ions viz.; Ag^+^, Na^+^, K^+^, Li^+^, Mn^2+^, Cd^2+^, Hg^2+^, Mg^2+^, Ca^2+^, Cr^3+^, Ni^2+^, Co^2+^, Cu^2+^, Zn^2+^, Pb^2+^, Fe^2+^and As^3+^ to the **L**_**1**_ solution results in no significant change in the absorbance and color (Fig. 2(b), Supplementary Fig. S4).

The emission studies of **L**_**1**_ at excitation wavelength 309 nm, shows weak fluorescence at 341 nm which is attributed to the isomerization of the imine bond (−HC=N) and excited state intramolecular proton transfer (ESIPT) from the −OH group to the imine nitrogen^30^. The titration of **L**_**1**_ with Al^3+^ shows gradual fluorescence enhancement with a bathochromic shift (red shift) of 2 nm having maximum fluorescence intensity at 343 nm. Also, emergence of two more emission peaks at 351 and 448 nm is observed (Fig. 2(c)).

The probable reason for the fluorescence switch on towards Al^3+^ may be due to (i) the photoinduced electron transfer (PET) suppressed by Al^3+^ coordination and (ii) new band in absorption spectrum^32^. PET shows changes in emission intensities with some or no spectral shifts. In our case, the spectral shift is of 2 nm in emission maxima during the fluorescence titration. The shorter fluorescence lifetime and lower quantum yield also support the operation of PET process^33^,^34^. The schematic representation of working mechanism is shown in Supplementary Fig. S5. Thus, the results obtained from absorption and emission studies validate that the sensing behavior of **L**_**1**_ is specific to Al^3+^ ions. The binding affinity of **L**_**1**_ with Al^3+^ is found to be 1.18 × 10^5 ^M^−1^ and 1.21 × 10^5 ^M^−1^ by absorption and emission methods, respectively by using Benesi-Hildebrand equation^35^,^36^ (Supplementary Fig. S6). The limit of detection (LOD) is found to be 86 nM. In general, chemosensor suffer with long response time^37^,^38^,^39^. In our case, the binding of Al(III) towards **L**_**1**_ is found to be very fast. After adding the aqueous solution of Al(III) to **L**_**1**_, the fluorescence intensity at 343 nm reached maximum within 120 s (Supplementary Fig. S7).

### Selectivity, reversibility and mode of binding

**Figure 3:**
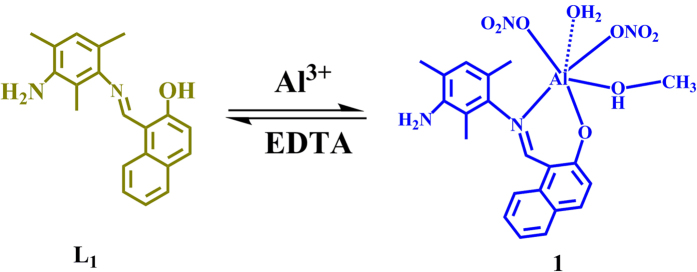
Schematic representation showing reversible nature of chemosensor L_1_.

Further, the selectivity test is performed using various metal ions with **L**_**1**_ which shows that only Al^3+^ ion exhibits fluorescence turn on (Fig. 2(d) and Supplementary Fig. S8(a)). A competitive selectivity test was also performed for various metal ions in presence of Al^3+^ in 1:4 ratio which shows that the presence of other metal ion do not affect the selective Al^3+^ sensing behavior of **L**_**1**_ (Supplementary Fig. S8(b)). The interference of DNA in the probe’s ability to detect Al(III) was examined and it was found that the DNA does not interfere with the sensing property of **L**_**1**_ (Supplementary Fig. S9). For validating the reversibility, the chemosensor **L**_**1**_ is studied by employing EDTA as a chelating agent. The increased fluorescence intensity of **L**_**1**_ with Al^3+^ ion was quenched by addition of EDTA (Fig. 3 and Supplementary Fig. S10).

Further, to understand the mode of complexation of **L**_**1**_ with Al^3+^ ion, ^1^H NMR titration is performed in DMSO-D6 and D_2_O solvents. On subsequent addition of Al^3+^ to **L**_**1**_, the napthyl –OH peak (15.34 ppm) decreases and finally at 0.8 eq. of Al^3+^, −OH peak was completely diminished which indicates complex formation of **L**_**1**_ with Al^3+^ (Supplementary Fig. S11). The stoichiometry of **1** is also confirmed by Job’s method which indicates 1:1 complex formation of **L**_**1**_ with **Al**^**3**+^ (Supplementary Fig. S12).

The optical properties of **1** results in high quantum yield (Φ = 0.172) with average life time <τ> = 0.99 ns. (Supplementary Figs S13, S14 and Supplementary Tables S3, S4). The performance comparison of **L**_**1**_ with other available fluorescent probes for Al^3+^ sensing is shown in Supplementary Table S5.

### Cytotoxicity assessment

To understand the practical ability of **L**_**1**_ and **1** as a sensor for biological systems, it is obvious to study the cytotoxicity. The cytotoxicity studies are carried out using MTT assay^40^. Human prostate cancer cell line DU145 is incubated with **L**_**1**_ and **1** (0.01–100 μM) for 24 h and the cell viability is quantified. The results summarized in Supplementary Fig. S15, indicates the cytocompatibility of the probe **L**_**1**_ and **1**. The cell viability is more than 80% at a high concentration of 100 μM which represent very low cytotoxicity of **L**_**1**_ and **1.**

### Intracellular Al^3+^ sensing

**Figure 4:**
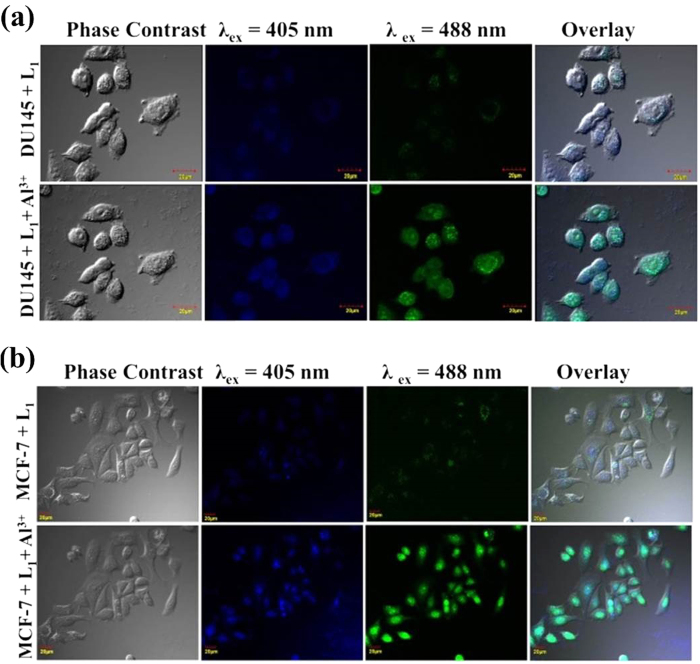
Intracellular Al^3+^ sensing in cancer cell lines (**a**) DU145 (**b**) MCF-7. (Excitation: 405 nm, Emission: 415–470 nm; Excitation: 488 nm, Emission: 500–550 nm).

The excellent sensing response and low cytotoxicity of **L**_**1**_, prompted us to explore for intracellular Al^3+^ sensing using confocal laser scanning microscopy. In this regard, two cell lines, DU145 and MCF-7 were first treated with 10 μM of **L**_**1**_ and incubated for 45 minutes at 37 °C. These **L**_**1**_ treated cells are subjected to confocal microscopy and were excited using lasers wavelength of 405nm and 488nm. As depicted in Fig. 4, the cells treated with **L**_**1**_resulted in a very weak intracellular fluorescence with blue and green colored signal at different excitation with uneven distribution in the cells. On addition of 10 μM of Al^3+^ to these cells, a considerable enhancement in blue and green colored fluorescent signal was observed. This confirms the ability of **L**_**1**_ to probe intracellular Al^3+^ and a possible intracellular multicolor bioimaging potential of **1**. Moreover, to our surprise, it is observed that the fluorescence signal becomes confined to the nuclei after some time (within 10 minutes), which hinted towards the possible nuclei specificity of **1**, which was further explored. The wavelength tuned emission spectra of **1** was recorded, which shows bathochromic shift with increase in excitation wavelength (Supplementary Fig. S16) resulting into multicolor bioimaging property. The similar wavelength tuned emission behavior of fluorophores is shown in previous reports^5^,^41^,^42^,^43^.

### Nucleus and nucleolus specific staining

**Figure 5:**
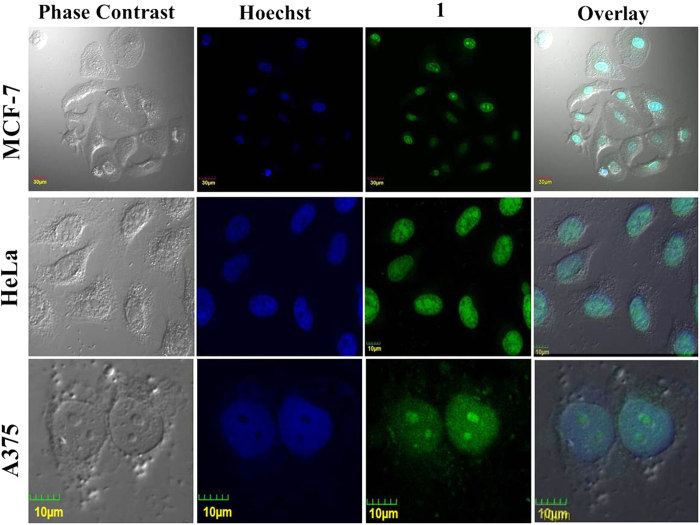
Nucleus specific staining using **1** in breast cancer cell line MCF-7, cervical cancer cell line HeLa and skin melanoma A375. (Excitation: 405 nm, Emission: 415–470 nm; Excitation: 488 nm, Emission: 500–550 nm).

The interesting intracellular sensing response by **L**_**1**_and hint of nucleus targeting by **1**, provoked us to study the bioimaging potential of **1.** The breast cancer cell line MCF-7, cervical cancer cell line HeLa and skin melanoma cell line A375 is used for comparison between the staining ability of a standard nucleus stain and **1**. These cells are first stained with Hoechst 33342 and the emission is recorded at λ_ex_ = 405 nm Further, 20 μM of **1** is added and after 5 minutes incubation, the intracellular emission is recorded at λ_ex_ = 488 nm. The blue fluorescence from nucleus specific Hoechst dye is completely overlapped by the nucleus staining green fluorescence of **1** (Fig. 5, Supplementary Fig. S17). The strong overlap validated the potential of **1** as a specific nucleus stain. The Pearson’s colocalisation coefficient was obtained to be R_r_ = 0.75 (Supplementary Fig. S18), which is in close agreement with recently reported nucleic acid selective probes^29^.

It is worth to mention that the dual staining experiment was performed in serial “staining and imaging” mode. First, the cells were stained with Hoechst and the fluorescence emission was recorded in blue channel in absence of **1** to avoid excitation of **1** in blue channel. Further, **1** was added without disturbing the cell position and the emission was recorded in green channel to avoid any interference with Hoechst fluorescence.

**Figure 6:**
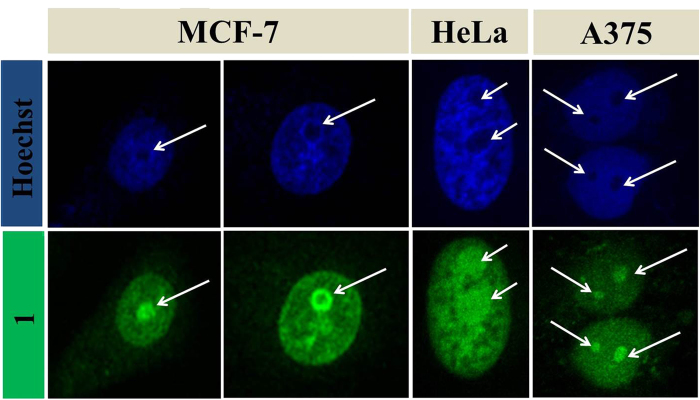
Nucleolus staining using **1** in breast cancer cell line MCF-7, cervical cancer cell line HeLa and skin melanoma A375. (Excitation: 405 nm, Emission: 415–470 nm; Excitation: 488 nm, Emission: 500–550 nm).

A closer look at the comparative nucleus stain of Hoechst and **1,** exhibits the dominance of **1** in not only staining nucleus but also nucleolus, which remains unstained in case of Hoechst as demonstrated in Fig. 6. To study intracellular photostability of **1,** the MCF-7 cells are stained with **1** and a dual channel emission video is recorded upto 450 scans, where laser of 405 and 488 nm is triggered at every scan (Supplementary Video 1). The video shows no obvious photobleaching, which suggests high photostability and feasibility of **1** as an efficient and suitable candidate for total nucleus stain. The fluorescence stability of **1** is also tested under continuous UV illumination up to two hours and varied temperature in range of (−80 °C to 90 °C), which shows high photostability of **1** (Supplementary Fig. S19(a,b)). The effect of pH values on the fluorescence intensity of **L**_**1**_ and **1** is also examined and shown in Supplementary Fig. S19(c,d)).

### DNase and RNase digestion test

**Figure 7:**
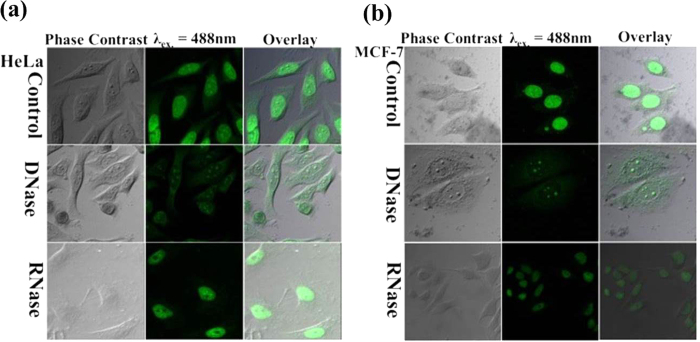
DNase and RNase digestion test in MCF-7, HeLa cell lines. (Excitation: 488 nm, Emission: 500–550 nm).

After exploring the specific nucleus and nucleolus staining capability of **1**, it is anticipated that **1** must specifically bind to nucleic acid. The mechanistic pathways and reason of specific nucleus and nucleolus staining by **1** is hypothesized by binding of **1** with DNA and RNA in the nucleus and nucleolus, respectively. The intracellular nucleic acid binding is explored by a deoxyribonuclease (DNase) and ribonuclease (RNase) digestion test^44^ and the results can be visualized in Fig. 7. HeLa and MCF-7 cell lines were stained with **1** and subsequently treated with DNase and RNase. Since, DNase hydrolyze only DNA substrate in the cells, the fluorescence of the nucleus is vanished while the nucleolus is found glowing in DNase treated cells, which confirmed the interaction of **1** with DNA present in the cell nucleus as evident in Fig. 7. Similarly, the RNase hydrolyze only the RNA substrate in the cells, resulting in no fluorescence from the nucleolus and the weak cytoplasmic fluorescence has also vanished while the nucleus was glowing in RNase treated cells, which approved the interaction of **1** with RNA present in the cells. The DNA binding of **1** is also validated by EtBr displacement assay and the results are shown in Supplementary Fig. S20. These observations supported the hypothesis of nucleic acid binding of **1** resulting into specific cellular localization and nucleus and nucleoli staining.

The photostability of **1** was studied in live cells and was compared with Hoechst and FITC. The intensity of FITC and Hoechst was reduced to 30% of the initial intensity after 1500 scans while the fluorescence from **1** was almost consistent and was clearly visible upto 3600 scans studied. (Supplementary Fig. S21).

The existence of RNA selective compounds is limited. One of the reasons is better affinity of small molecules towards double stranded DNA than single stranded RNA^45^. Also, the complicated environment of living cells, sometimes results in non-specific binding and imaging. SYTO RNA-Select is the only commercially available live cell RNA staining dye. Another existing limitation comes from photo-bleaching and self–quenching of nucleus and nucleolus staining agents. The present work successfully overcomes these limitations and reports a nucleic acid specific compound which stains both the nucleus and the nucleolus with better photostability.

## Discussion

In summary, we for the first time report the specific staining of nucleus and nucleoli by **1**, which is prepared in a facile manner. The ligand **L**_**1**_ involved in synthesis of **1** also promotes selective and sensitive sensing of Al^3+^. **1** is explored for specific nucleic acid binding using DNA and RNA digestion and employed as a specific nucleus and nucleolus staining agent. Our results are expected to inspire replacement of existing expensive and sensitive nucleic acid specific dyes. Use of **1** for both nucleus and nucleoli staining can be of great scientific interest.

## Methods

### Synthesis of chemosensor (L_1_)

2,4,6-trimethylbenzene-1,3-diamine (0.189 g, 1mmol) dissolved in dry methanol (10ml) was added to a solution of 2-Hydroxy-1-napthaldehyde (0.172 g, 1mmol) in methanol (15ml). The content of the flask heated under reflux for 3 h. A yellow oily compound was formed which is monitored by TLC. Solvent was evaporated on *vacuo* and washed with hexane. A yellow solid was obtained which was dried under vacuum. Needle shaped yellow crystals were suitable for single crystal X-ray analyses were obtained by recrystallization from methanol-chloroform (2:1) within two days. Characterization data. ^1^H NMR (400 MHz, CDCl_3_): δ15.27(s, 1H), 8.96(s, 1H, −CH=N), 7.92(d, J = 8Hz, 1H), 7.80(d, J = 8Hz, 1H), 7.71(d, J = 8Hz, 1H), 7.45(t, J = 8Hz, 1H), 7.30(t, J = 8Hz, 1H), 7.45(s, 1H), 7.11(d, J = 8Hz.1H), 6.89(s, 1H), 3.(s, 2H), 2.20(s, 3H), 2.19(s, 3H), 2.13(s, 3H).). ^13^C NMR (400 MHz, CDCl3): δ170.10, 160.67, 143.05, 141.51, 136.25, 133.36, 129.86, 129.25, 128.00, 127.04, 123.22, 122.36, 119.74, 119.08, 118.51, 114.19, 108.4, 17.98, 17.45, 12.60. IR (KBr, cm^−1^): 3447(br), 2360(w), 1620(vs), 1532(w), 1475(w), 1311(w), 1093(w), 838(w), 746(w). λ_max_ = 310, 360 nm, HRMS (m/z): [M] calcd. for C_20_H_20_N_2_O, 305.16; found, 305.13; analysis (calcd., found for C_20_H_20_N_2_O): C(77.57, 78.92), H(6.73, 6.62), N(9.85, 9.20).

### Synthesis of 1

A methanolic solution (10 mL) of Al(NO_3_)_2_.9H_2_O (0.375g 1mmol) was added dropwise with stirring to a solution of **L**_**1**_ (0.304g 1mmol) in methanol (10 mL). The reaction mixture was stirred for additional 6 h, washed with Diethyl ether twice and evaporated on *vacuo*. Characterization data: ^27^Al NMR (400 MHz, DMSO-D6): δ 3.34; HRMS (*m/z*): [M] calcd. for C_21_H_25_AlN_4_O_9_, 503.43; found, 502.98; λ_max_ = 419, 360 and 309 nm.

### Nucleus specific staining

Breast cancer cell line MCF-7, cervical cancer cell line HeLa and skin melanoma A375 were seeded in confocal dishes. Adherent cells were stained with Hoescht 33342 (50μg/mL) and incubated for 15 minutes at at 37 °C in 5% CO_2_. The cells were rinsed with PBS thrice and was visualised under confocal microscope at an excitation of λ_ex_. = 405nm and λ_ex_. = 488nm. Without changing the positioning of cells under microscope, cells were stained with **1** (30 μM) and kept for 10 minutes. Further the cells were visualised using confocal microscope at λ_ex_. = 488nm.

### DNase and RNase digestion tests

MCF-7 and HeLa cell lines were first seeded in confocal dishes. The cells were fixed by cold methanol (HPLC grade) for 1 min. Further, the cells were treated with 1% Triton X-100 to make the cell membrane permeabilized. The cells were rinsed thrice with 1X PBS to remove the leftover detergent. Afterwards, cells were stained with 30 μM **1**. After staining cells were rinsed with PBS thrice. Blank PBS (as control experiment), 50 mg/ml DNase or 75 mg/ml RNase was added into different confocal dishes containing cells and kept in an incubator at 37 °C in 5% CO_2_ for 3 hr. Further, the cells were subjected to confocal microscopy.

### Physical measurements

^1^H NMR (400 MHz), and^13^C NMR(100 MHz) spectra were recorded on the Bruker Avance (III) instrument by using CDCl_3_.^1^H NMR chemical shifts are reported in parts per million (ppm) relative to the solvent residual peak (CDCl_3_, 7.26 ppm). ^13^C NMR chemical shifts are reported relative to the solvent residual peak (CDCl_3_, 77.36 ppm). IR spectra [4000−400 cm^−1^] were recorded with a Bio-Rad FTS 3000MX instrument on KBr pellets. Elemental analyses were carried out with a Thermo-Flash 2000 elemental analyser. Spectrophotometric measurement were performed on a varian UV-vis spectrophotometer (model: Cary 100) using a quartz cuvette with a path length of 1cm. The excitation and emission slits were 5/5 nm for the emission measurements. The mass spectra were recorded on Brucker-Daltonics, micrOTOF-QII mass spectrometer. The life time measurement recorded on TCSPC system from Horiba Yovin (Model: Fluorocube-01-NL). The samples were excited at 375 nm using a picosecond diode laser (Model: Pico Brite-375L) with the repetition rate of 5 MHz. The signals were collected at magic angle (54.70) polarization using a photomultiplier tube (TBX-07C) as detector, which has a dark counts less than 20 cps. The instrument response function of our setup was 150 ps. The data analysis was performed using IBH DAS (version 6, HORIBA Scientific, Edison, NJ) decay analysis software. NMR spectra of **L**_**1**_ and **1** are depicted in Supplementary Figs S22–S25.

## Additional Information

**How to cite this article**: Saini, A. K. *et al*. The development of fluorescence turn-on probe for Al(III) sensing and live cell nucleus-nucleoli staining. *Sci. Rep*. **6**, 34807; doi: 10.1038/srep34807 (2016).

## Supplementary Material

Supplementary Information

Supplementary Video S1

## References

[b1] Rivera-Fuentes P. & Lippard S. J. Metal-Based Optical Probes for Live Cell Imaging of Nitroxyl (HNO). Acc. Chem. Res. 48, 2927–2934 (2015). 26550842 10.1021/acs.accounts.5b00388

[b2] Carter K. P., Young A. M. & Palmer A. E. Fluorescent Sensors for Measuring Metal Ions in Living Systems. Chem. Rev. 114, 4564–4601 (2014). 24588137 10.1021/cr400546e PMC4096685

[b3] Chyan W., Zhang D. Y., Lippard S. J. & Radford R. J. Reaction-based fluorescent sensor for investigating mobile Zn^2+^ in mitochondria of healthy versus cancerous prostate cells. Proc. Natl. Acad. Sci. 111, 143–148 (2014). 24335702 10.1073/pnas.1310583110 PMC3890804

[b4] Xu R. . Nanoscale Metal–Organic Frameworks for Ratiometric Oxygen Sensing in Live Cells. J. Am. Chem. Soc. 138, 2158–2161 (2016). 26864385 10.1021/jacs.5b13458 PMC5139906

[b5] Sharma V., Saini A. K. & Mobin S. M. Multicolour fluorescent carbon nanoparticle probes for live cell imaging and dual palladium and mercury sensors. J. Mater. Chem. B 4, 2466–2476 (2016). 10.1039/c6tb00238b 32263196

[b6] Kim S. . Salicylimine-Based Fluorescent Chemosensor for Aluminum Ions and Application to Bioimaging. Inorg. Chem. 51, 3597–3602 (2012). 22385332 10.1021/ic2024583

[b7] Fasman G. D. Aluminum and Alzheimer’s disease: model studies. Coord. Chem. Rev. 149, 125–165 (1996).

[b8] Nayak P. Aluminum: Impacts and Disease. Environ. Res. 89, 101–115 (2002). 12123643 10.1006/enrs.2002.4352

[b9] Cronan C. S., Walker W. J. & Bloom P. R. Predicting aqueous aluminium concentrations in natural waters. Nature 324, 140–143 (1986).

[b10] Berthon G. Aluminium speciation in relation to aluminium bioavailability, metabolism and toxicity. Coord. Chem. Rev. 228, 319–341 (2002).

[b11] Valeur B. & Leray I. Design principles of fluorescent molecular sensors for cation recognition. Coord. Chem. Rev. 205, 3–40 (2000).

[b12] Gupta V. K., Shoora S. K., Kumawat L. K. & Jain A. K. A highly selective colorimetric and turn-on fluorescent chemosensor based on 1-(2-pyridylazo)-2-naphthol for the detection of aluminium(III) ions. Sens. Actuators B Chem. 209, 15–24 (2015).

[b13] Sen S. . A water soluble Al3+ selective colorimetric and fluorescent turn-on chemosensor and its application in living cell imaging. Analyst 137, 3975–3981 (2012). 22785321 10.1039/c2an35560d

[b14] Liu S. . A multifunctional phosphorescent iridium(III) complex for specific nucleus staining and hypoxia monitoring. Chem. Commun. 51, 7943–7946 (2015). 10.1039/c5cc01978h 25866315

[b15] Gill M. R. . A ruthenium(II) polypyridyl complex for direct imaging of DNA structure in living cells. Nat. Chem. 1, 662–667 (2009). 21378959 10.1038/nchem.406

[b16] Kang Y.-F., Fang Y.-W., Li Y.-H., Li W. & Yin X.-B. Nucleus-staining with biomolecule-mimicking nitrogen-doped carbon dots prepared by a fast neutralization heat strategy. Chem. Commun. 51, 16956–16959 (2015). 10.1039/c5cc06304c 26445735

[b17] Wang X. . Steering graphene quantum dots in living cells: lighting up the nucleolus. J. Mater. Chem. B 4, 779–784 (2016). 10.1039/c5tb02474a 32262959

[b18] Maity D. & Govindaraju T. Pyrrolidine constrained bipyridyl-dansyl click fluoroionophore as selective Al^3+^ sensor. Chem. Commun. 46, 4499–4501 (2010). 10.1039/c0cc00119h 20480098

[b19] Wang Y.-W. . A colorimetric and fluorescent turn-on chemosensor for Al^3+^ and its application in bioimaging. Tetrahedron Lett. 50, 6169–6172 (2009).

[b20] Samanta S., Goswami S., Hoque M. N., Ramesh A. & Das G. An aggregation-induced emission (AIE) active probe renders Al(III) sensing and tracking of subsequent interaction with DNA. Chem. Commun. 50, 11833–11836 (2014). 10.1039/c4cc05093b 25145610

[b21] Boyer P. D. . Delivering Single-Walled Carbon Nanotubes to the Nucleus Using Engineered Nuclear Protein Domains. ACS Appl. Mater. Interfaces 8, 3524–3534 (2016). 26783632 10.1021/acsami.5b12602

[b22] Kong W. . High-bright fluorescent carbon dots and their application in selective nucleoli staining. J. Mater. Chem. B 2, 5077–5082 (2014). 10.1039/c4tb00579a 32261841

[b23] Ai J. . Multifunctional near-infrared fluorescent nanoclusters for simultaneous targeted cancer imaging and photodynamic therapy. Sens. Actuators B Chem. 222, 918–922 (2016).

[b24] Liu W. . Deep-Red Emissive Crescent-Shaped Fluorescent Dyes: Substituent Effect on Live Cell Imaging. ACS Appl. Mater. Interfaces 7, 7421–7427 (2015). 25785397 10.1021/acsami.5b01429

[b25] Zhou B. . Imaging of nucleolar RNA in living cells using a highly photostable deep-red fluorescent probe. Biosens. Bioelectron. 68, 189–196 (2015). 25569876 10.1016/j.bios.2014.12.055

[b26] Yu J., Parker D., Pal R., Poole R. A. & Cann M. J. A Europium Complex That Selectively Stains Nucleoli of Cells. J. Am. Chem. Soc. 128, 2294–2299 (2006). 16478184 10.1021/ja056303g

[b27] Zhang K. Y. . Structure, Photophysical and Electrochemical Properties, Biomolecular Interactions, and Intracellular Uptake of Luminescent Cyclometalated Iridium(III) Dipyridoquinoxaline Complexes. Inorg. Chem. 49, 2530–2540 (2010). 20131874 10.1021/ic902465b

[b28] Shen R. . Multifunctional Conjugates To Prepare Nucleolar-Targeting CdS Quantum Dots. J. Am. Chem. Soc. 132, 8627–8634 (2010). 20518506 10.1021/ja1002668

[b29] Li D. . Nucleic acid-selective light-up fluorescent biosensors for ratiometric two-photon imaging of the viscosity of live cells and tissues. Chem. Sci. 7, 2257–2263 (2016). 10.1039/c5sc03956h PMC5977445 29910915

[b30] Saini A. K., Srivastava M., Sharma V., Mishra V. & Mobin S. M. A highly selective, sensitive and reversible fluorescence chemosensor for Zn^2+^ and its cell viability. Dalton Trans. 45, 3927–3935 (2016). 26831322 10.1039/c5dt04945h

[b31] Mobin S. M., Mishra V. & Chaudhary A. Isolation of a Metastable Intermediate in a Heterometallic CuII–HgII 1D Polymeric Chain: Synthesis, Crystal Structure, and Photophysical Properties. Inorg. Chem. 54, 1293–1299 (2015). 25615821 10.1021/ic502514e

[b32] Kwon J. E. . Fluorescent Zinc Sensor with Minimized Proton-Induced Interferences: Photophysical Mechanism for Fluorescence Turn-On Response and Detection of Endogenous Free Zinc Ions. Inorg. Chem. 51, 8760–8774 (2012). 22534151 10.1021/ic300476e

[b33] Ashokkumar P., Ramakrishnan V. T. & Ramamurthy P. Photoinduced Electron Transfer (PET) Based Zn2+ Fluorescent Probe: Transformation of Turn-On Sensors into Ratiometric Ones with Dual Emission in Acetonitrile. J. Phys. Chem. A 115, 14292–14299 (2011). 22066705 10.1021/jp209061f

[b34] Zhang J. F., Zhou Y., Yoon J. & Kim J. S. Recent progress in fluorescent and colorimetric chemosensors for detection of precious metal ions (silver, gold and platinum ions). Chem. Soc. Rev. 40, 3416–3429 (2011). 21491036 10.1039/c1cs15028f

[b35] Benesi H. A. & Hildebrand J. H. A Spectrophotometric Investigation of the Interaction of Iodine with Aromatic Hydrocarbons. J. Am. Chem. Soc. 71, 2703–2707 (1949).

[b36] Shiraishi Y., Sumiya S., Kohno Y. & Hirai T. A Rhodamine−Cyclen Conjugate as a Highly Sensitive and Selective Fluorescent Chemosensor for Hg(II). J. Org. Chem. 73, 8571–8574 (2008). 18828632 10.1021/jo8012447

[b37] Rajamalli P. & Prasad E. Low Molecular Weight Fluorescent Organogel for Fluoride Ion Detection. Org. Lett. 13, 3714–3717 (2011). 21688866 10.1021/ol201325j

[b38] Lin Q. . Rationally designed anion-responsive-organogels: sensing F−via reversible color changes in gel–gel states with specific selectivity. Soft Matter 10, 5715–5723 (2014). 24985608 10.1039/c4sm00841c

[b39] Lin Q. . A highly selective and sensitive fluorescence ‘turn-on’ fluoride ion sensor. RSC Adv. 5, 11786–11790 (2015).

[b40] Carmichael J., DeGraff W. G., Gazdar A. F., Minna J. D. & Mitchell J. B. Evaluation of a tetrazolium-based semiautomated colorimetric assay: assessment of chemosensitivity testing. Cancer Res. 47, 936–942 (1987). 3802100

[b41] Wallrabe H. & Periasamy A. Imaging protein molecules using FRET and FLIM microscopy. Curr. Opin. Biotechnol. 16, 19–27 (2005). 15722011 10.1016/j.copbio.2004.12.002

[b42] Suhling K., French P. M. W. & Phillips D. Time-resolved fluorescence microscopy. Photochem. Photobiol. Sci. 4, 13–22 (2005). 15616687 10.1039/b412924p

[b43] wang L. & Zhou H. S. Green synthesis of luminescent nitrogen-doped carbon dots from milk and its imaging application. Anal. Chem. 86, 8902–8905 (2014). 25181643 10.1021/ac502646x

[b44] Lu Y.-J. . A molecular fluorescent dye for specific staining and imaging of RNA in live cells: a novel ligand integration from classical thiazole orange and styryl compounds. Chem. Commun. 51, 15241–15244 (2015). 10.1039/c5cc05551b 26329127

[b45] Chang Y. T., Li Q. & Rosania G. inventors; New York University, assignee. RNA-selective styryl probes for live cell imaging of nuclear structure and function. United States patent US 7,790,896. Sept 7, 2010.

