# Supplementary material for: The development of fluorescence turn-on probe for Al(III) sensing and live cell nucleus-nucleoli staining
Source: Sci Rep. 2016 Oct 10;6:34807. doi: 10.1038/srep34807 (PMC5056391; doi:10.1038/srep34807)
Supplement: Supplementary Information [file srep34807-s1.doc]

**Supplementary Information**

**The development of fluorescence turn-on probe for Al(III) sensing and live cell nucleus-nucleoli staining**

Anoop Kumar Saini,^1^ Vinay Sharma,^2^ Pradeep Mathur*^1^ & Shaikh M. Mobin*^1,2,3^

^1^Discipline of Chemistry, Indian Institute of Technology Indore, Simrol, Indore 453552, India.

^2^Centre for Biosciences and Bio-Medical Engineering, Indian Institute of Technology Indore, Simrol, Indore 453552, India.

^3^Metallurgical Engineering and Material Science, Indian Institute of Technology Indore, Simrol Indore 453552, India.

Email: [xray@iiti.ac.in](mailto:xray@iiti.ac.in) director@iiti.ac.in

**Supplementary Fig**. **S1**. Synthesis of **H_2_L^1^**


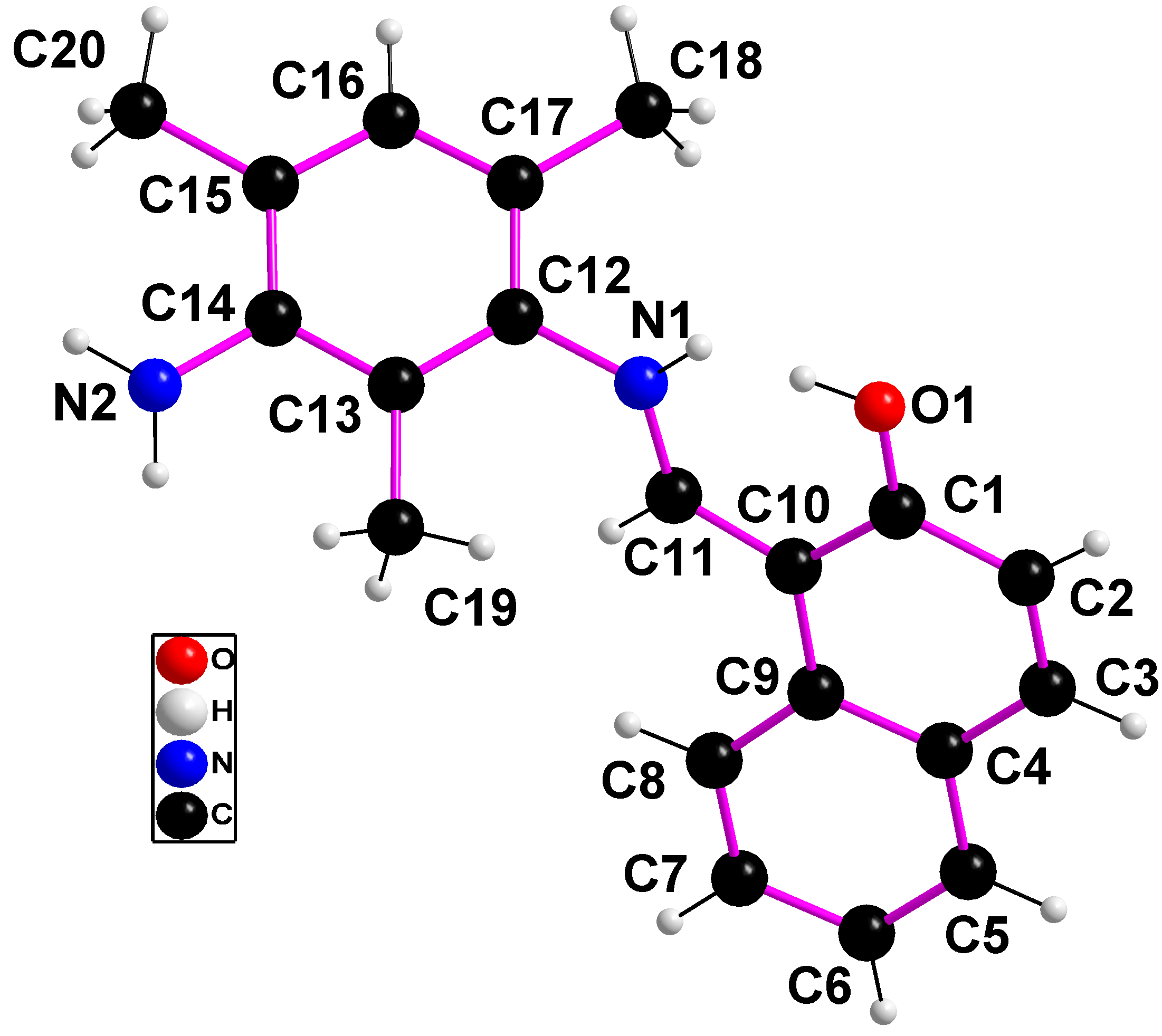


**Supplementary Fig**. **S2.** Molecular structure of **L_1_**


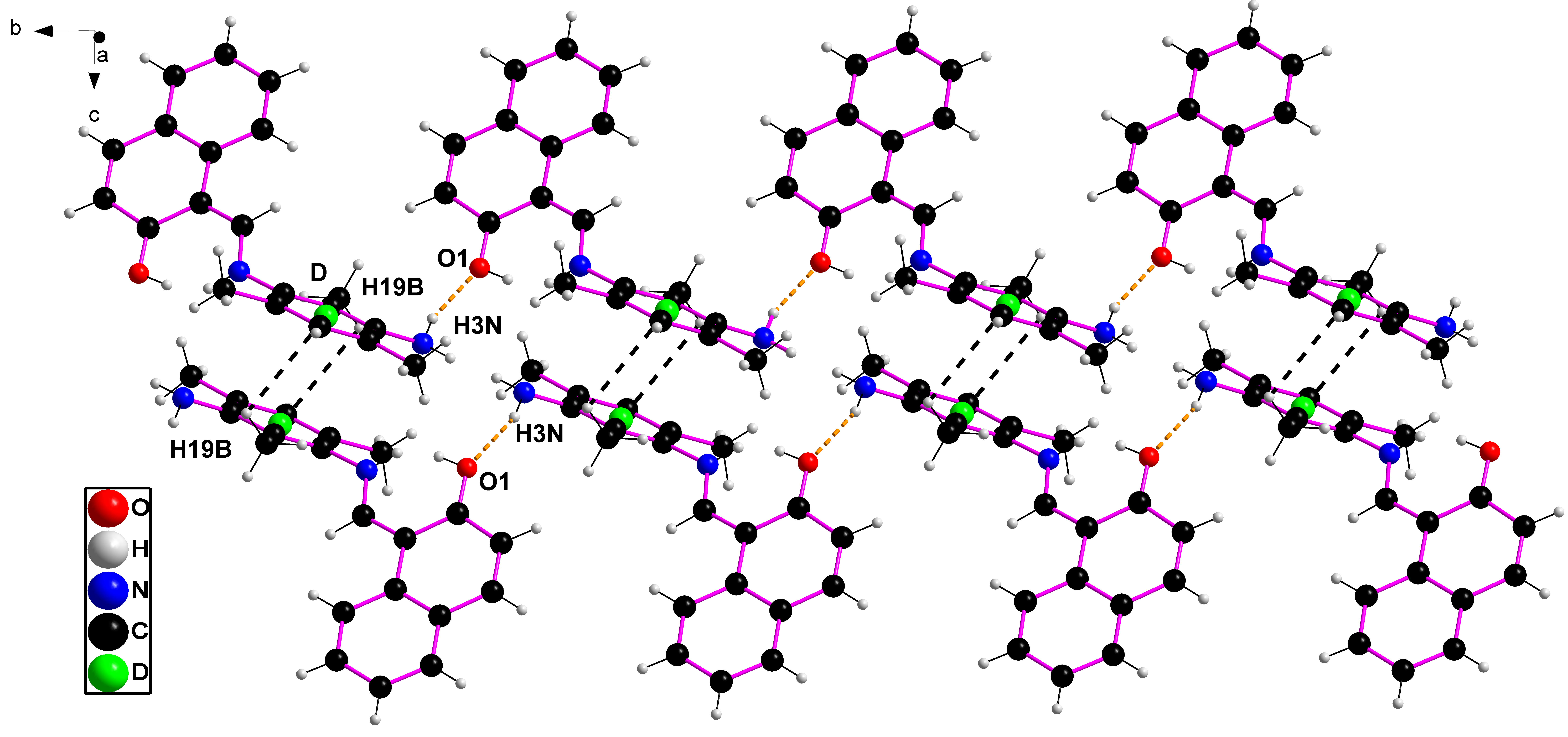


**Supplementary Fig**. **S3**. Packing diagram of **L_1_,** where D is the dummy atom.


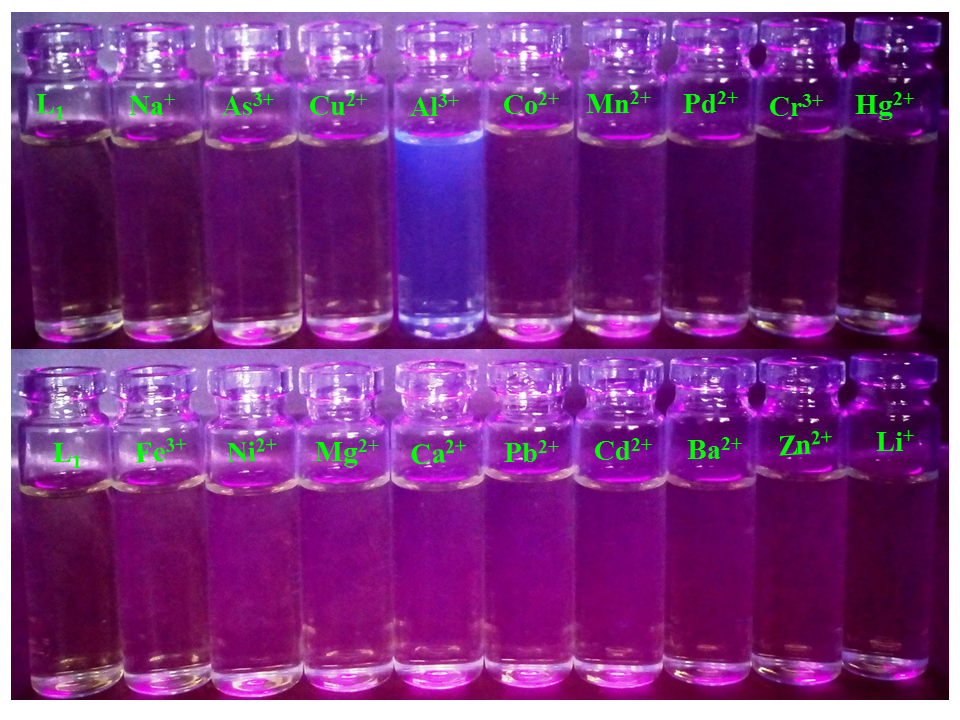


**Supplementary Fig**. **S4**. Colorimetric detection of **Al^3+^** using **L_1_** (10 equivalent of each metal ions with respect to **L_1_**).

**
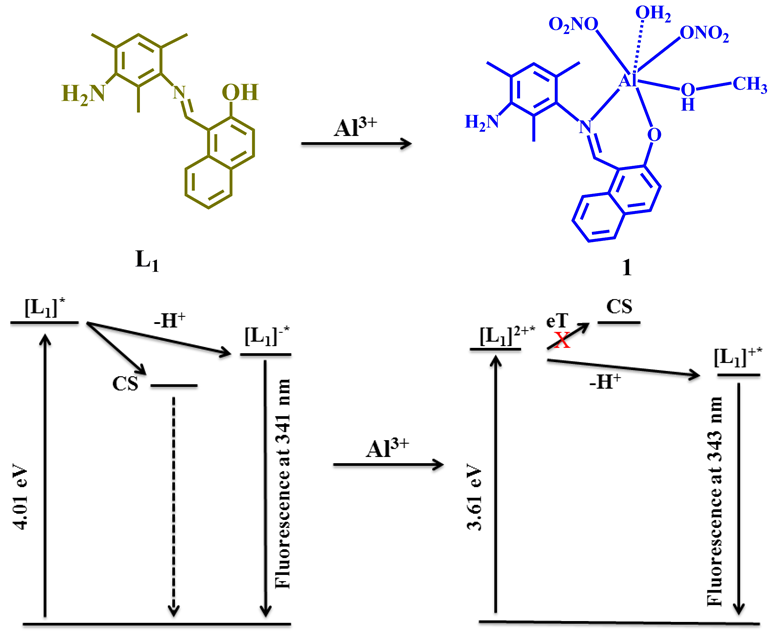
**

**Supplementary Fig**. **S5**. Schematic representation of PET process: **[L_1_]*,** Frank-Condon excited state; CS, an intramolecular charge-separated state; **[L_1_]^2^*,** Frank-Condon excited state of **L_1_-Al** ; **[L_1_]***, Frank-Condon excited state of a deprotonated complex **1**.


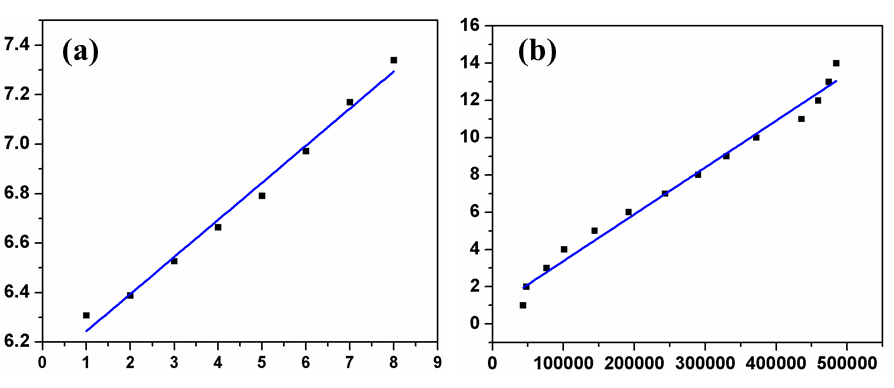


**Supplementary Fig**. **S6**. **(a)** Bensei- Hildbrand plot(B-H plot) obtained from absorption (at 419 nm wavelength) studies. Binding constant (1.18×10^5^M^-1^) curve of sensor **L_1_** with Al^3+^ determined by UV-visible spectroscopy. **(b)** B-H plot obtained from Fluorescence (at 343 nm emission wavelength) studies. Binding constant (1.21×10^5^M^-1^) curve of sensor **L_1_** with Al^3+^ determined by fluorescence spectroscopy.


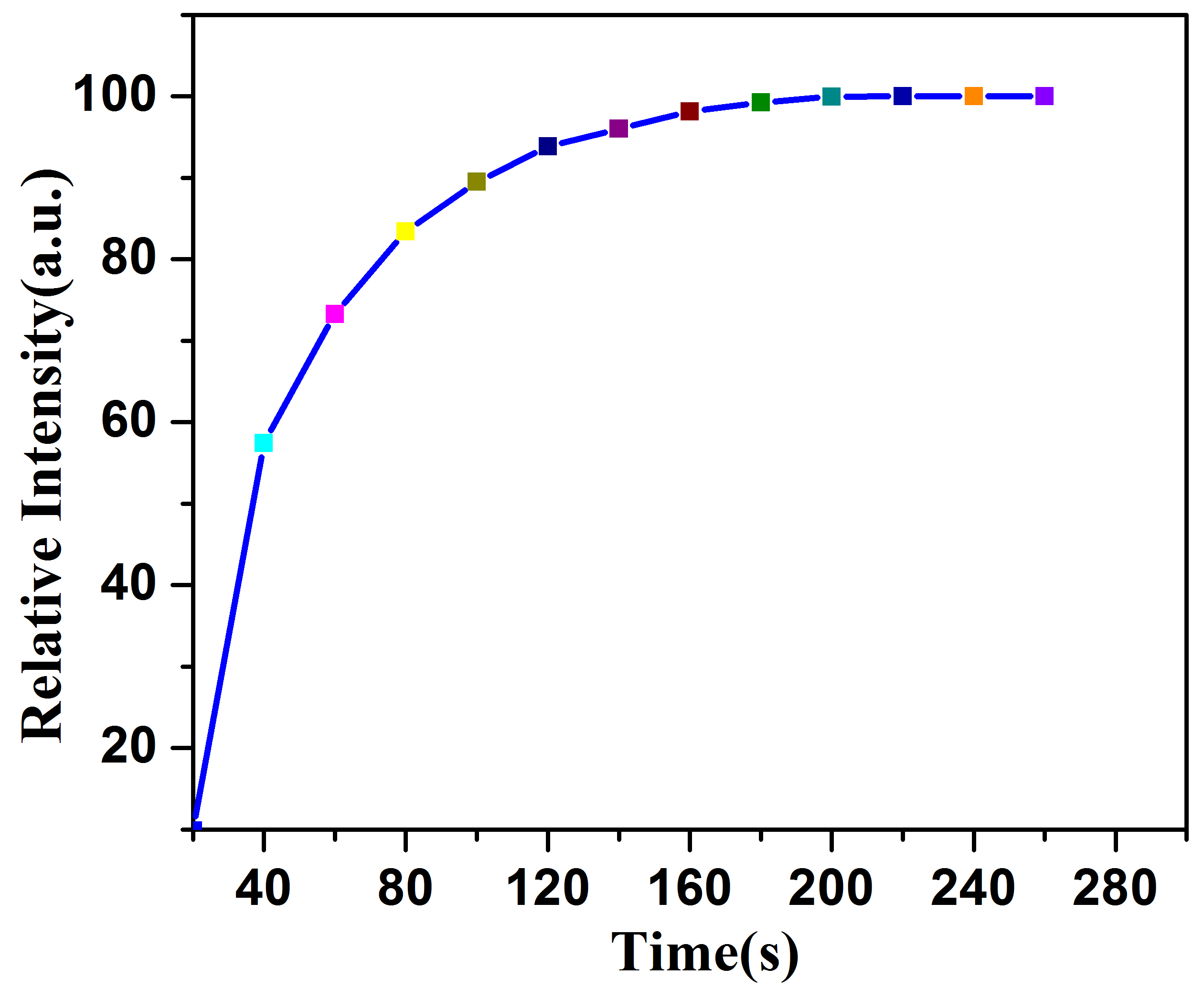


**Supplementary Fig**. **S7**. Fluorescence intensity at 343 nm for **L_1_** (3.0×10^-4^) in MeOH after addition of Al(III) (1.0×10^-3^) in aqueous solution.


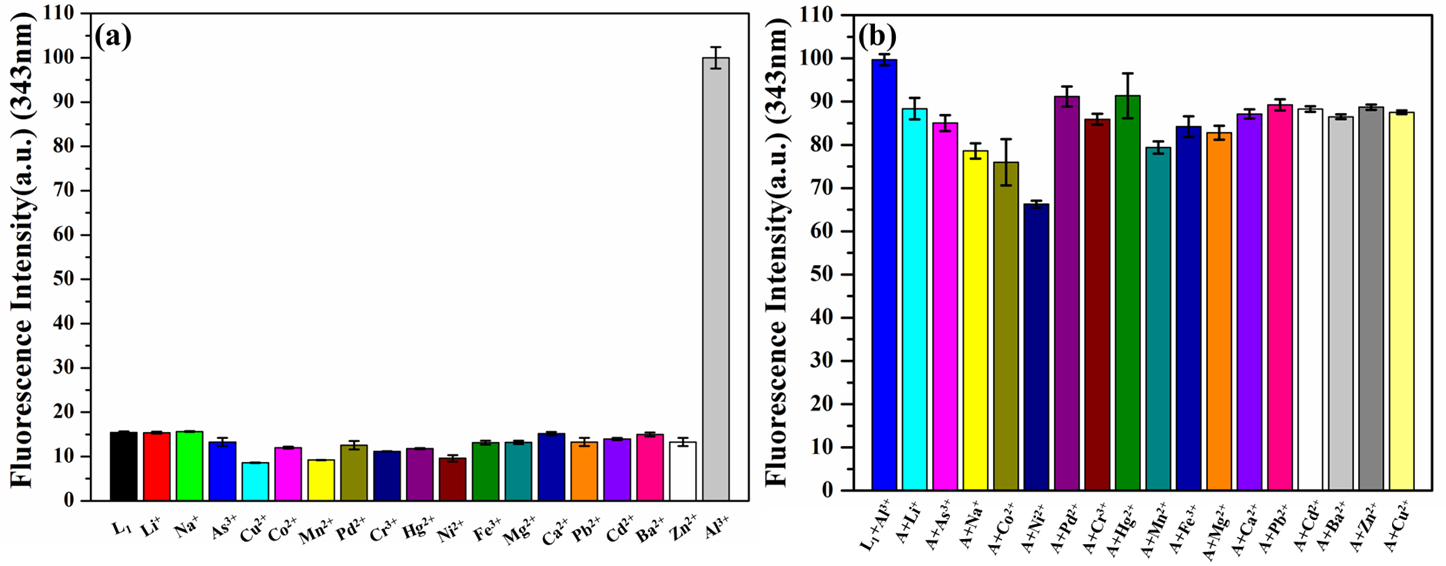


**Supplementary Fig**. S**8**. (**a**) Histogram showing the relative fluorescence response of various metal ions (c=1.0**×**10^-3^M) with **L_1_** (c=1.0**×**10^-5^ M) in aq. ACN (ACN/H_2_O = 7:3 v/v, 10µM HEPES buffer, pH = 7.4. (**b**) The effect of influence of other metal ions in the presence of Al^3+^ ions and **L_1_** (A= **L_1_**+ Al^3+^). (Data: Mean±SD)

**
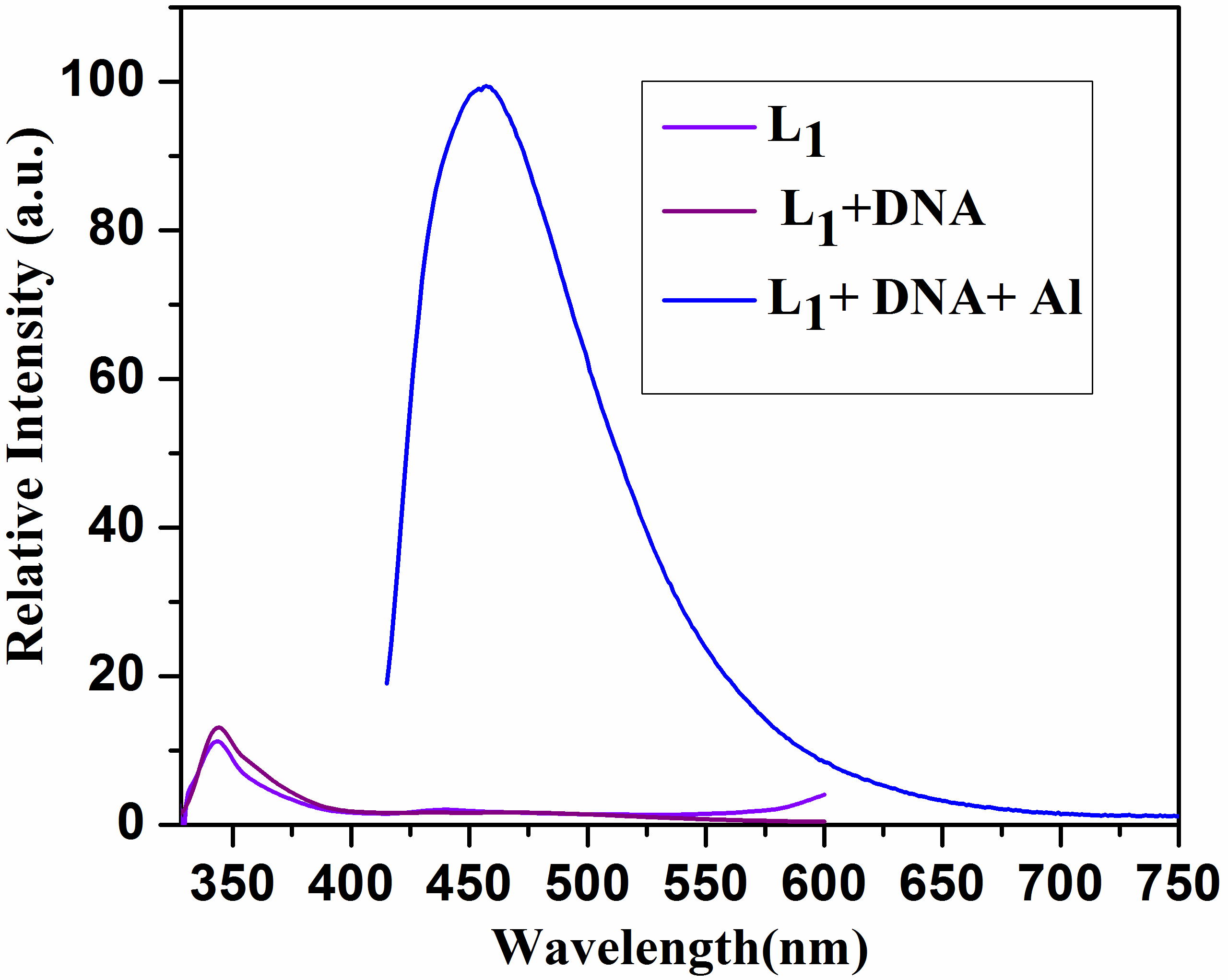
**

**Supplementary Fig**. **S9**. Effect of DNA towards the sensing response of **L_1_**.


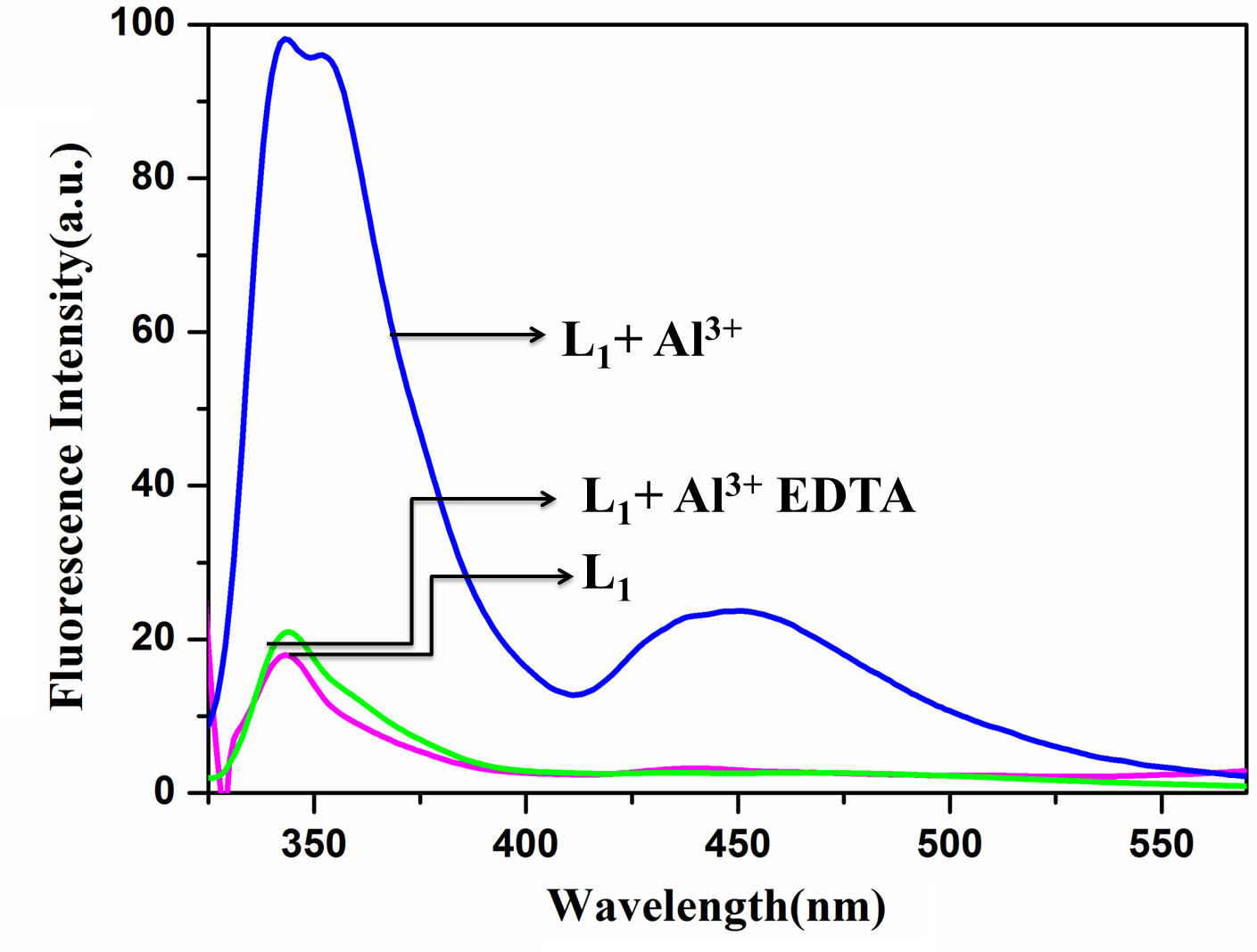


**Supplementary Fig**. **S10**. Reversible nature of chemosensor **L_1_**.


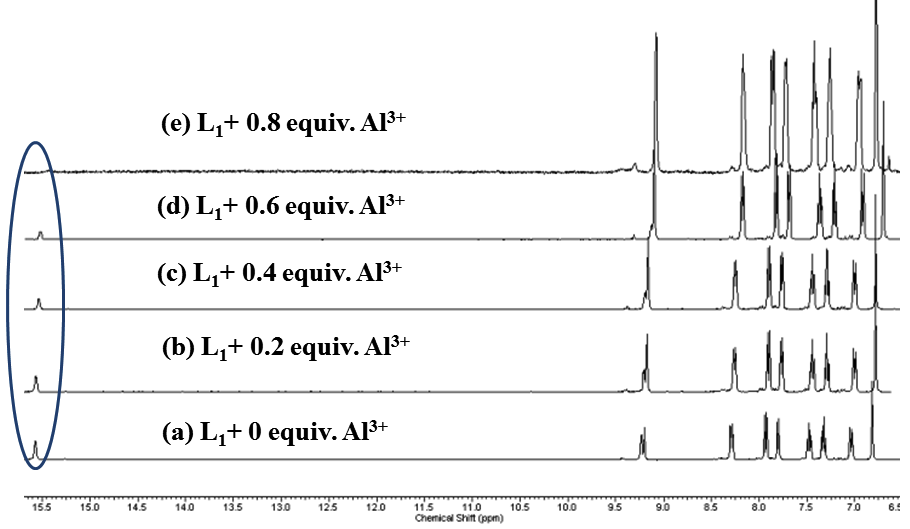


**Supplementary Fig**. **S11**. **^1^H** NMR Spectra (400MHz) of **L_1_** in DMSO-D6 at 25˚C and the corresponding changes after the gradual addition of different equivalents of Al(NO_3_)_2_.9H_2_O in D_2_O (**a**) **L_1_**, (**b**) **L_1_ +** 0.2 equiv. Al^3+^, (**c**) **L_1_ +** 0.4 equiv. Al^3+^ ,(**d**) **L_1_ +** 0.6 equiv. Al^3+^, (**e**) **L_1_+** 0.8 equiv. Al^3+^.


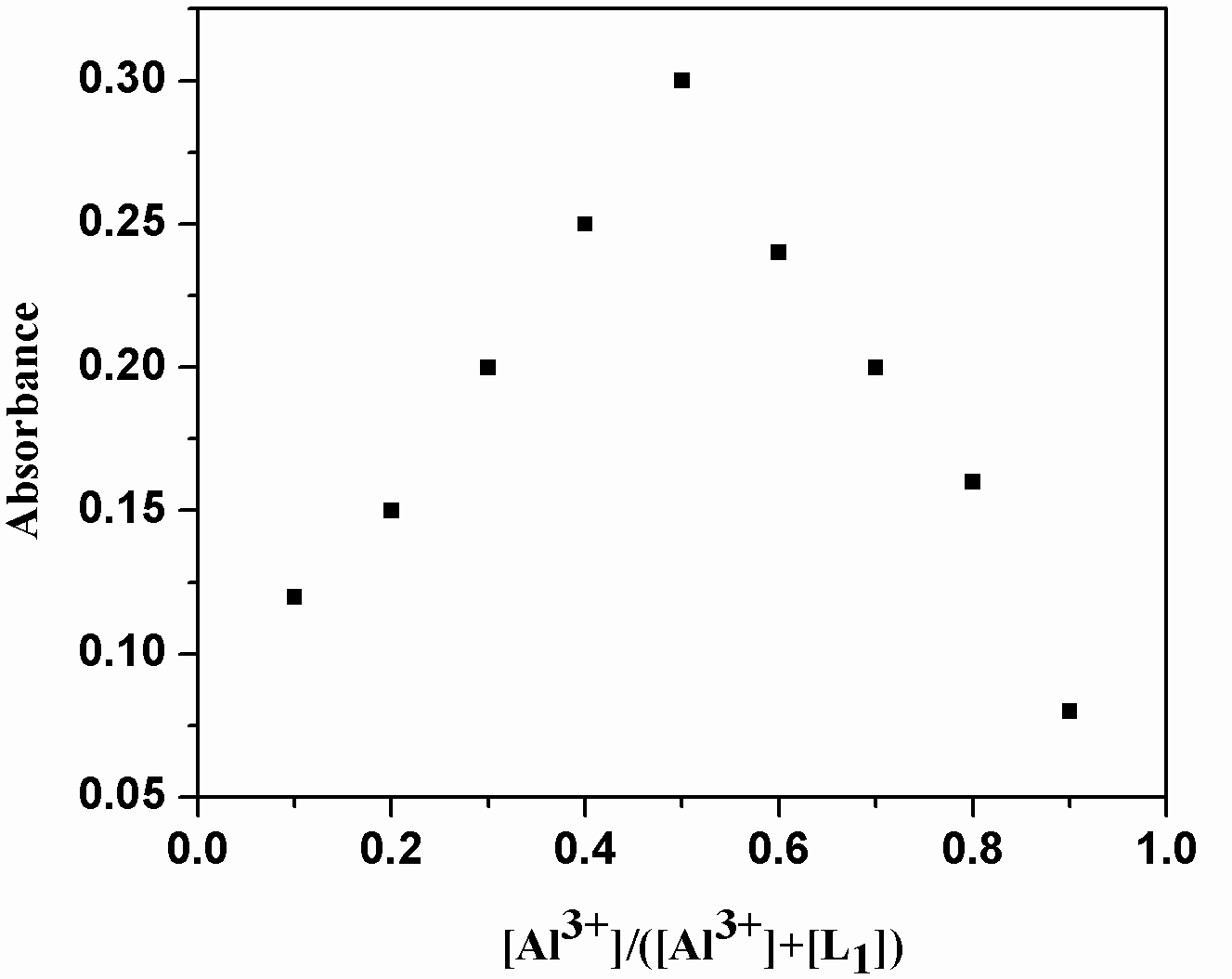


**Supplementary Fig**. S**12**. Job’s plot


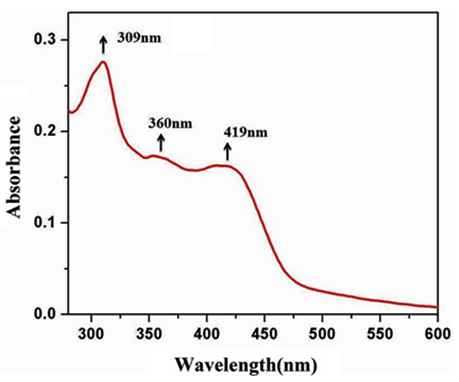


**Supplementary Fig**. **S13**. Absorption spectra of **1** in MeOH (c=1.0×10^-5^M).


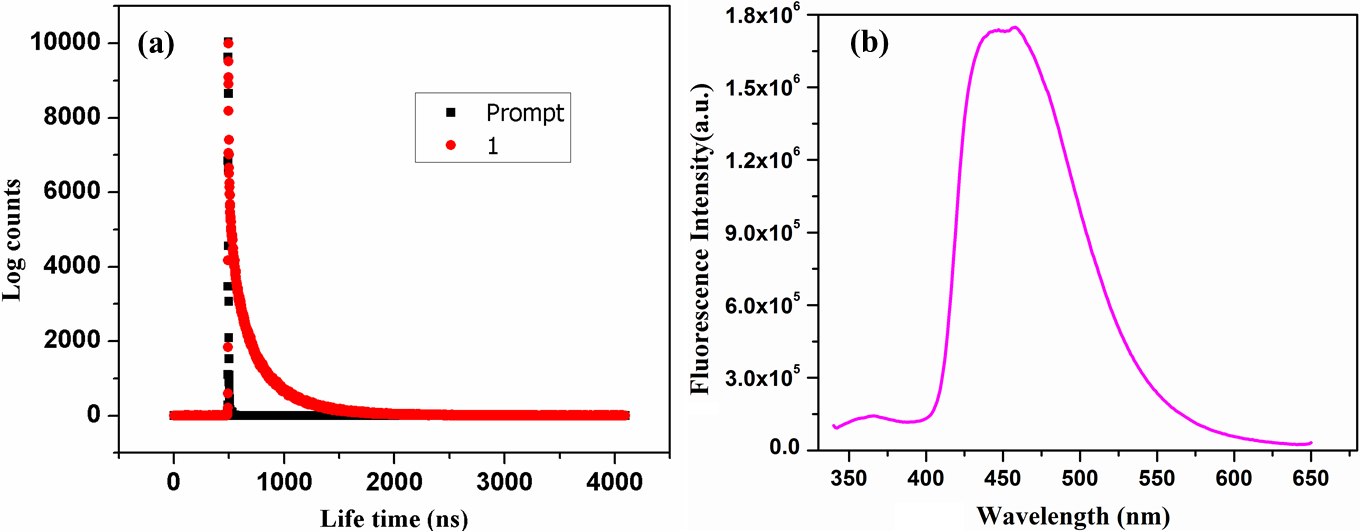


**Supplementary Fig**. **S14**. (**a**) Average life time measurement of **1** in MeOH (λ_ex_ =375 nm) (**b**) Fluorescence spectra of **1** in MeOH.


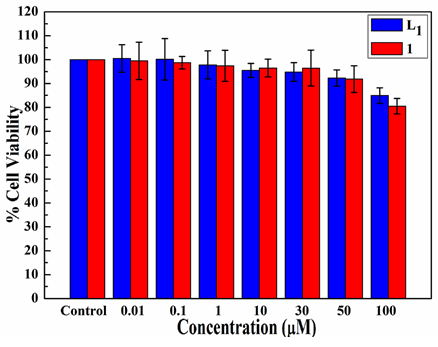


**Supplementary Fig**. **S15**. Cell viability profile of **L_1_** and **1** against prostate cancer line DU145

**
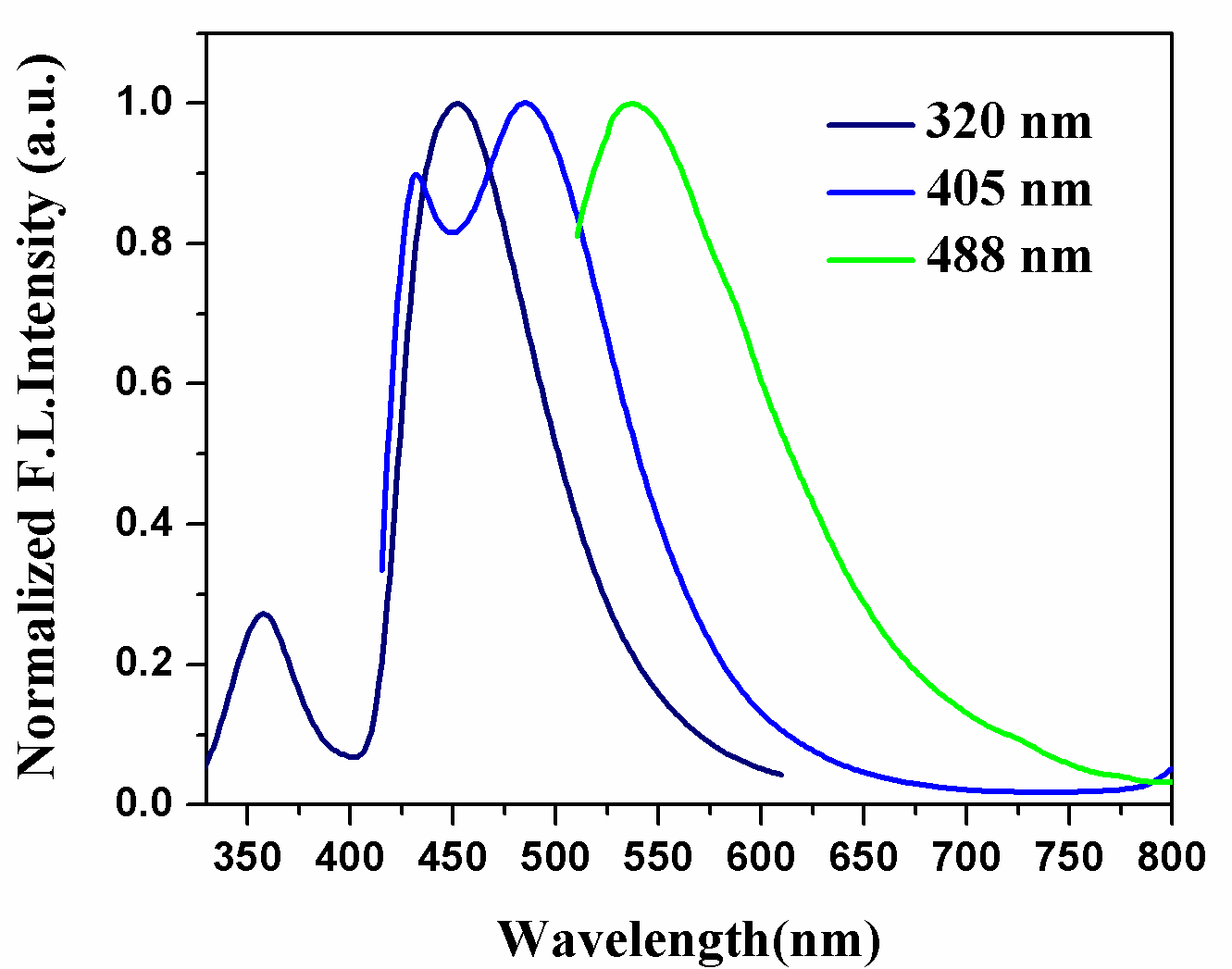
**

**Supplementary Fig**. **S16**. Emission spectra of **1** at different excitation wavelengths in MeOH.

**(A)**

**
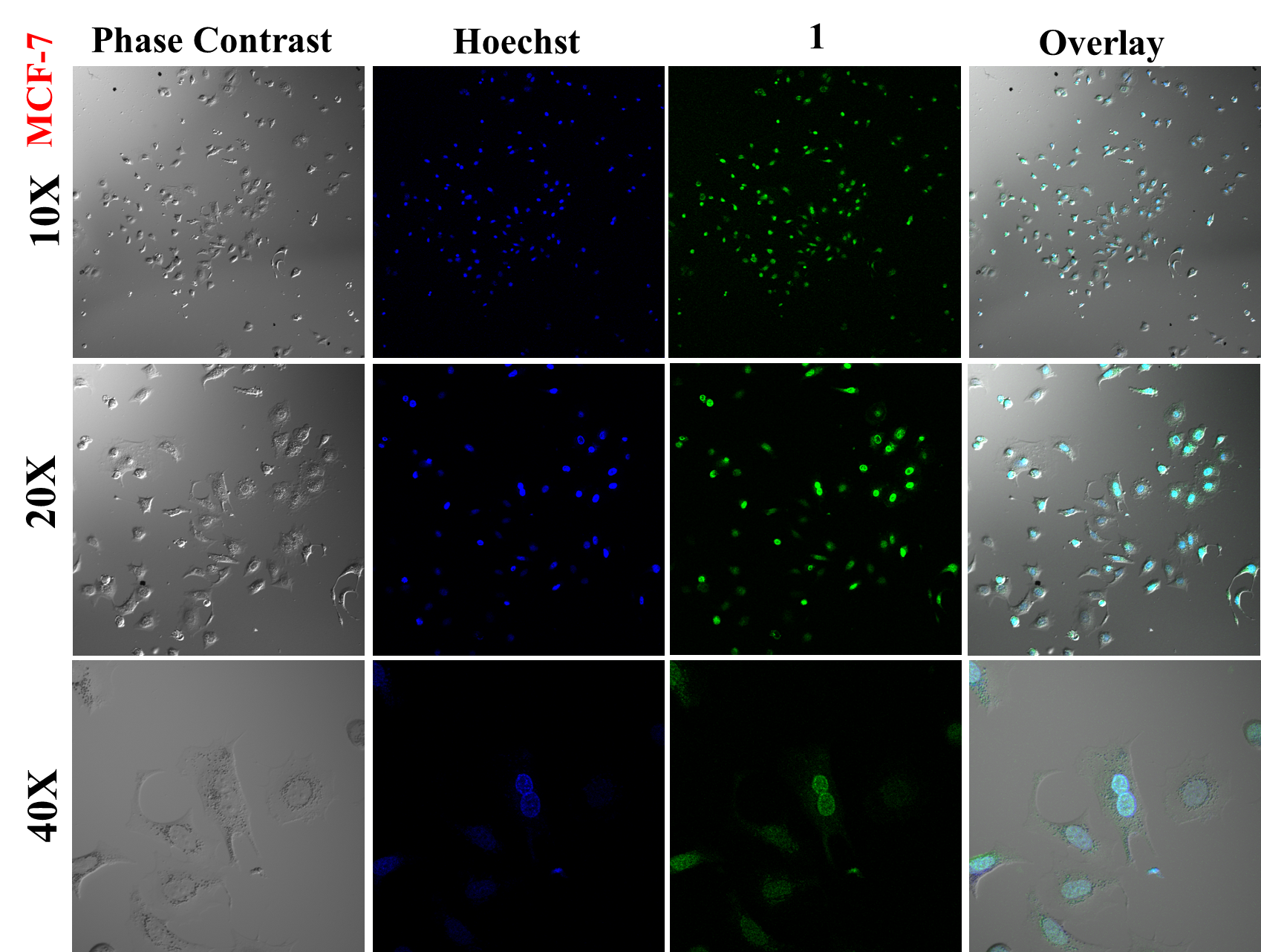
**

**(B)**

**
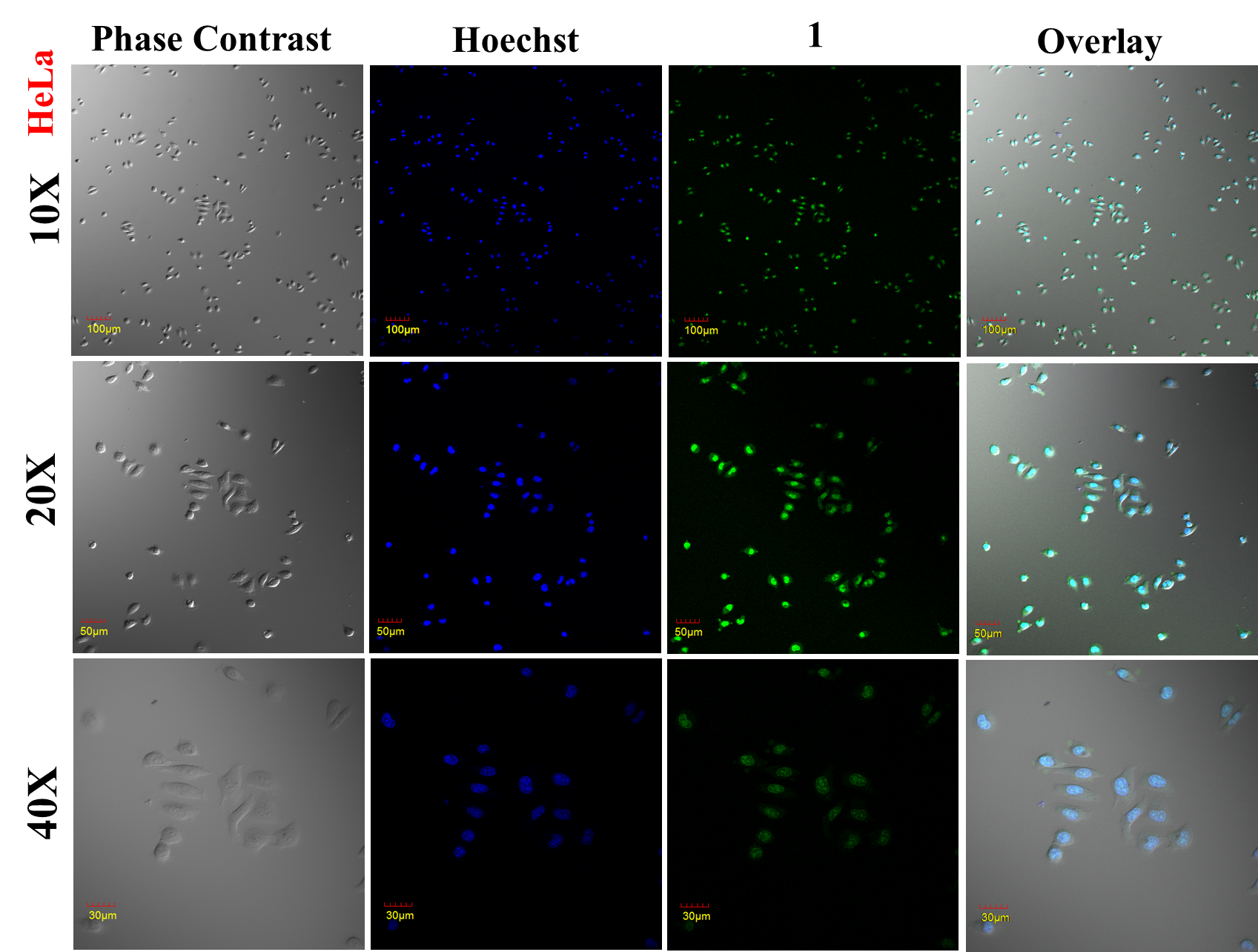
**

**Supplementary Fig**. **S17**. Nucleus specific staining of **1**. (**A**) in **MCF-7** cell line and (**B**) In **HeLa** cell line.

**
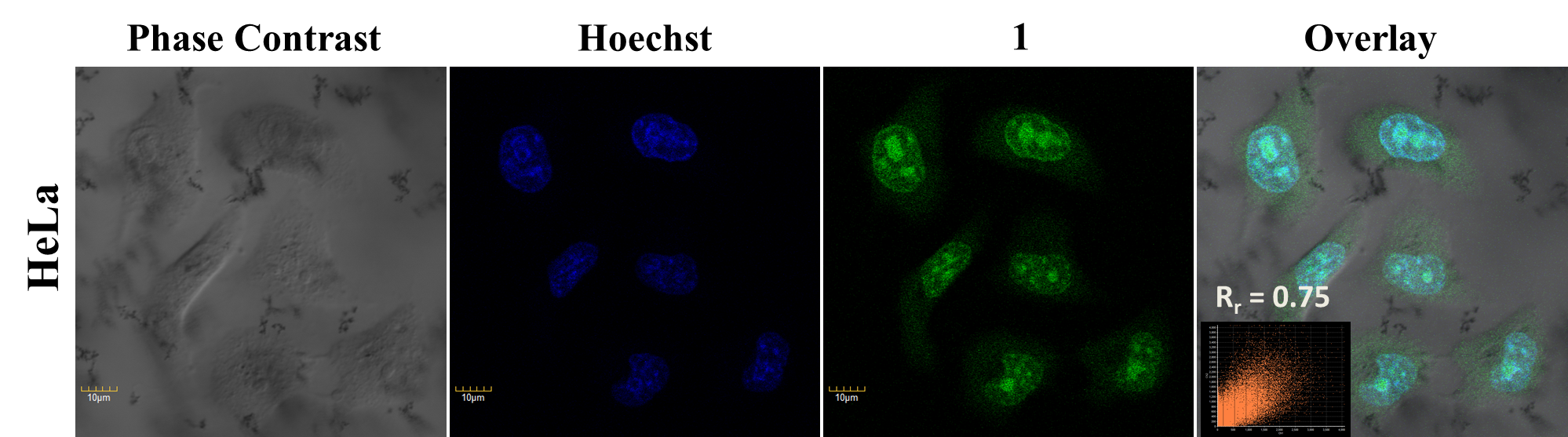
**

**Supplementary Fig**. **S18**. Pearson’s colocalisation coefficient for nucleus specificity of **1**.

**Supplementary Video SV1**. Photo stability of **1** in live cells


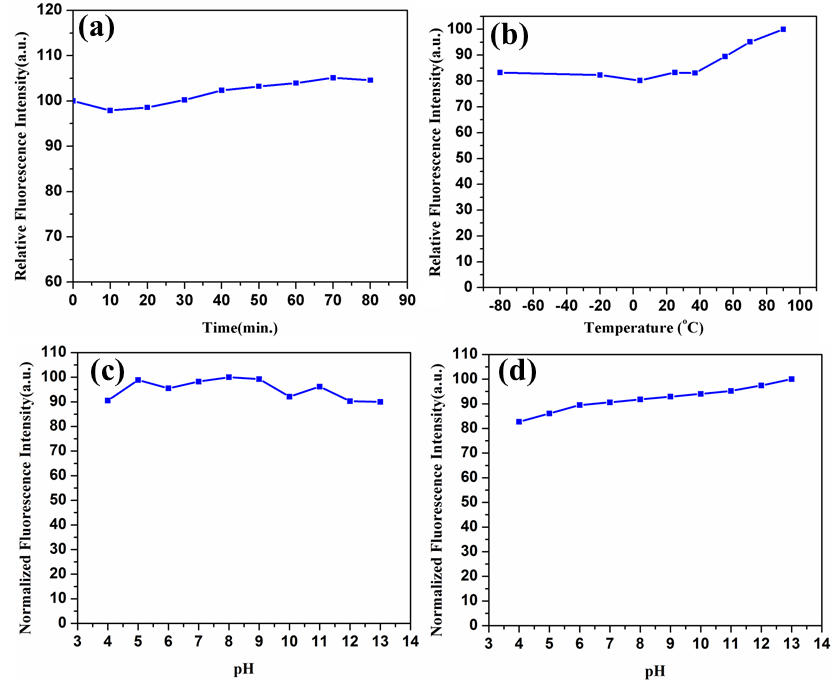


**Supplementary Fig. S19**. Stability of fluorescence intensity at (**a**) varied UV illumination (**1**), (**b**) varied temperature (**1**), (c) varied pH (**1**) and (d) varied pH (**L_1_**)

**
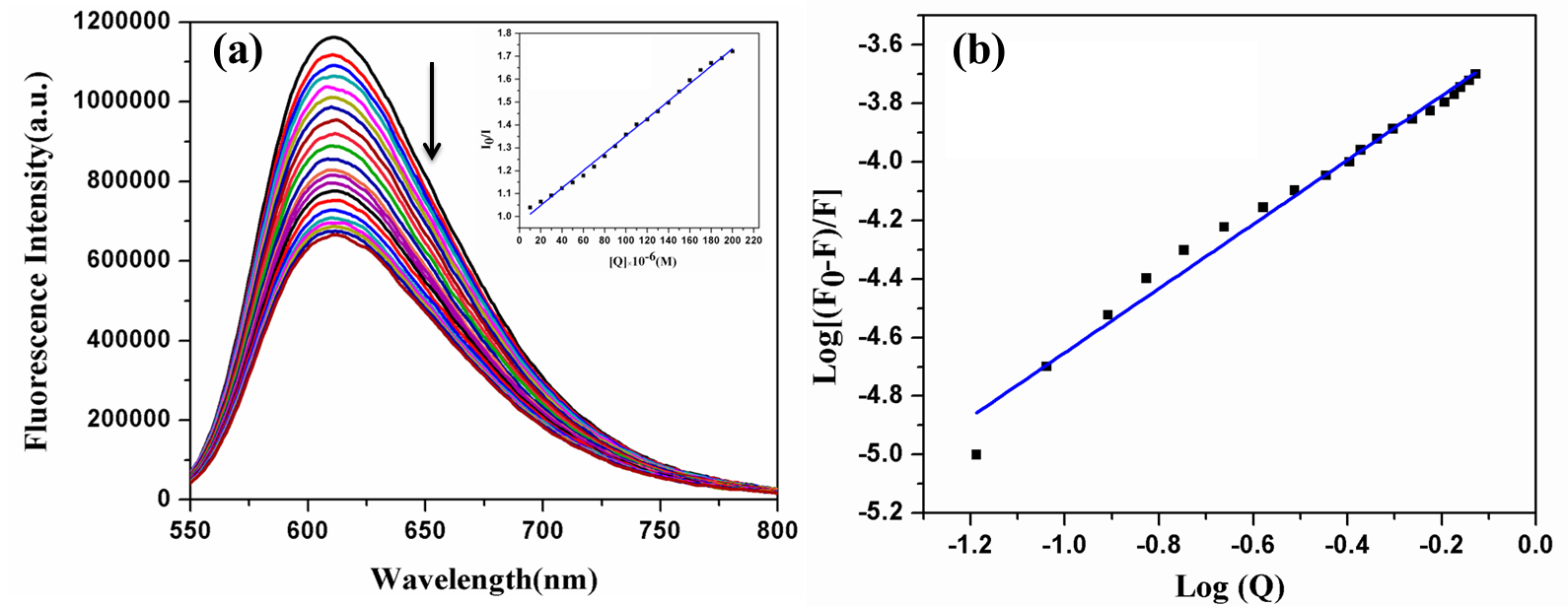
**

**Supplementary Fig**. **S20**. (a) EtBr displacement assay by change in fluorescence intensity of EB bound DNA with increasing conc. of **1** Inset: corresponding stern-volmer plot. **(b)** Scatchard plot for determination of K_a_.

**(A)**

**
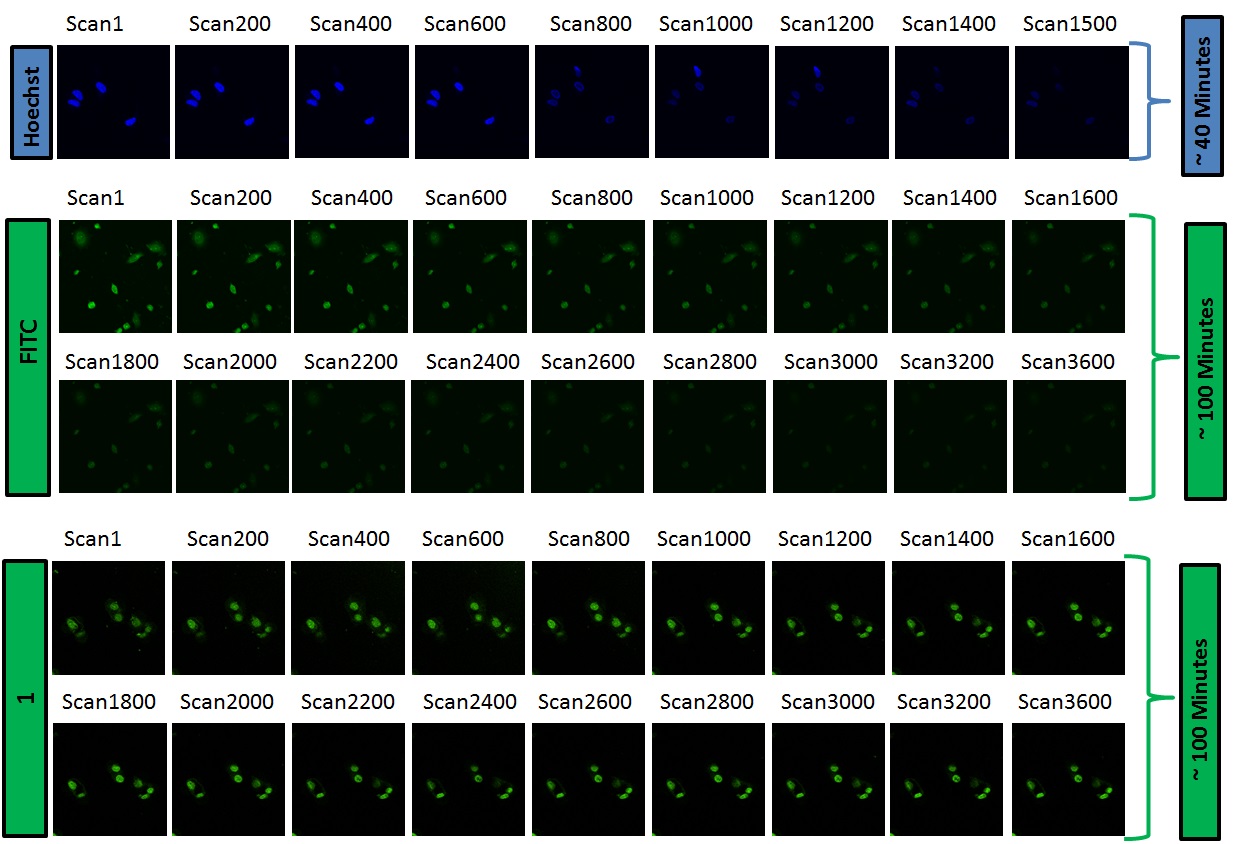
**

**(B)**

**
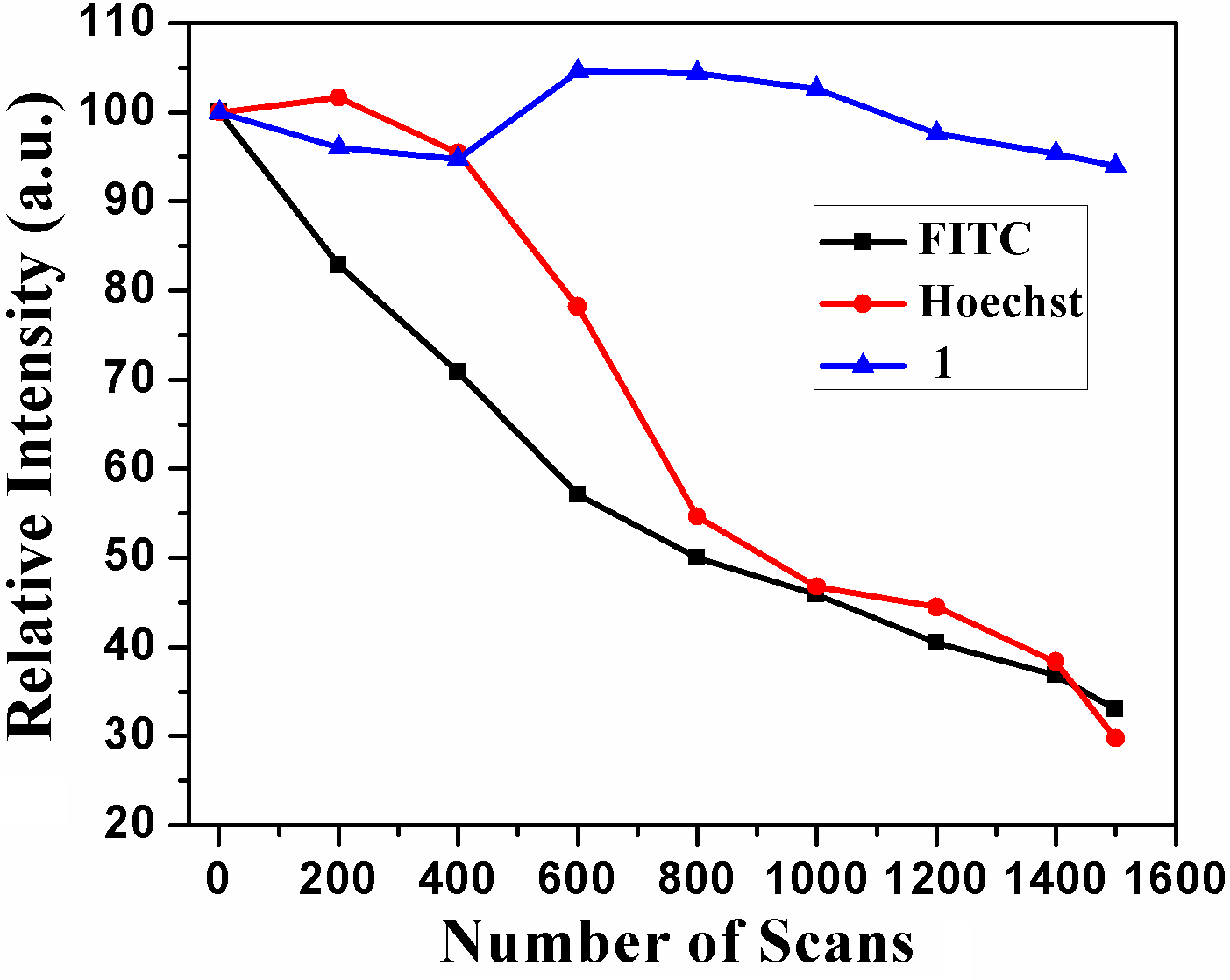
**

**Supplementary Fig**. **S21**. Comparative *In vitro* photostability of FTIC, Hoechst and **1** in MCF-7 cell lines.

**
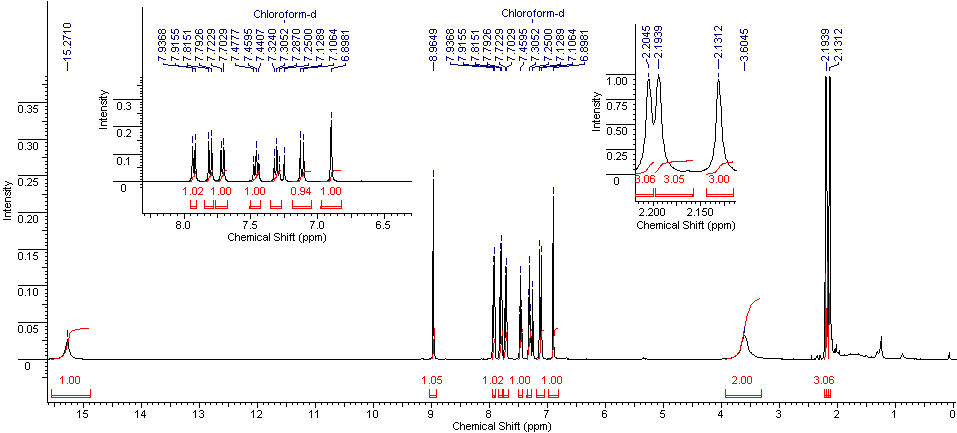
**

**Supplementary Fig**. **S22**.^1^H NMR spectrum of **L_1_**

**Supplementary Fig**. **S23**.^13^C NMR spectrum of **L_1_**

**
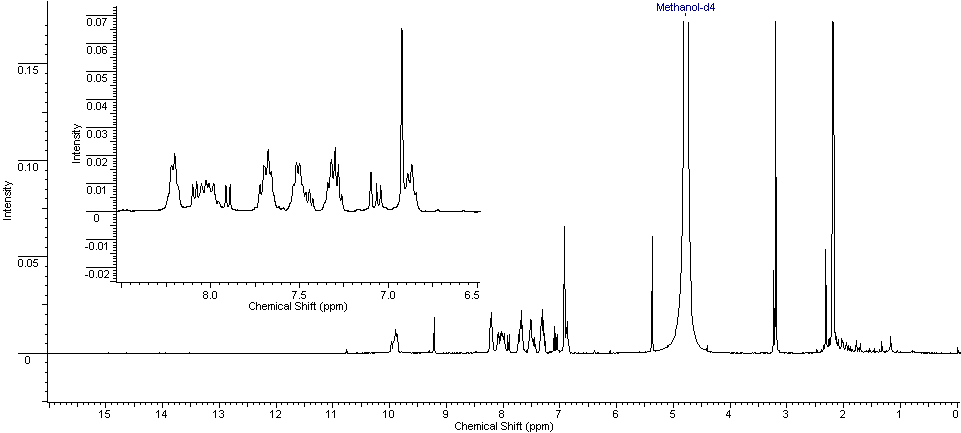
**

**Supplementary Fig**. **S24**.^1^H NMR spectrum of **1**

**
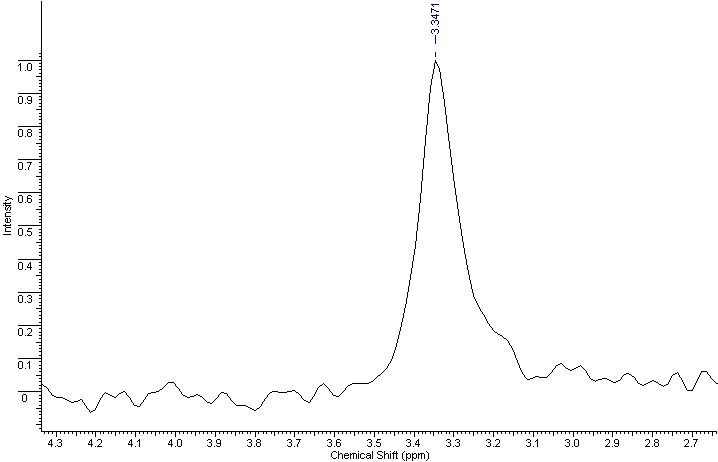
**

**Supplementary Fig**. **S25**.^27^Al NMR spectrum of **1**

**Supplementary Table S1.** Crystallographic parameters of **L_1_**

| **Identification code** | **L_1_** (293K) |
| --- | --- |
| Empirical formula | C_20_H_21_N_2_O_1_ |
| Formula weight | 305.39 |
| Temperature | 293(2) K |
| Wavelength | 1.5418 A |
| Crystal system, space group | Monoclinic, *P*2_1_/*n* |
| Unit Cell Parameter |  |
| *a/*Å | 8.3362(2) |
| *b*/Å | 8.4186(2) |
| *c/*Å | 23.0992(5) |
| α/^o^ | 90 |
| β/^o^ | 92.636(2) |
| γ/^o^ | 90 |
| *V*/Å^3^ | 1619.37(6) |
| *Z*, *d*_calcd_(mg/m^3^) | 4, 1.253 |
| μ/ mm_-1_ | 0.608 |
| *F*(000) | 652 |
| θ range | 3.831 to 71.287 |
| Index ranges | -10<=h<=10,  -9<=k<=10,  -28<=l<=24 |
| Reflections collected / unique | 10180 / 3123 [R(int) = 0.0217] |
| Data / restraints / parameters | 3123 / 0 / 219 |
| GOF, F^2^ | 1.060 |
| R1, wR2 [I>2σ(I)] | R1 = 0.0490, wR2 = 0.1383 |
| R1, wR2 (all data) | R1 = 0.0578, wR2 = 0.1468 |
| CCDC | 1453041 |

**Supplementary Table S2**. Hydrogen Bonding in **L_1_ [**Å and (**_o_**)].

|  | D-H...A | d(D-H) | d(H...A) | d(D...A) | <(DHA) |
| --- | --- | --- | --- | --- | --- |
| **L_1_** | | | | | |
| **1** | N(2)-H(3)N…O(1)#(2) | 0.923(.000) | 2.155(.001) | 3.048(.001) | 162.50( 0.04) |
| **Equivalent positions:**  ( 2) x,+y+1,+z | | | | | |

**Supplementary Table S3**. Photophysical properties of **L_1_** and **1**

| Compound^a^ λ_(S0→S1)_ (nm) ε (M^−1^ cm^−1^) λ_em_^b^ (nm) Φ^C^ |
| --- |

**L_1_** 310 2.9 × 10^3^

360 1.9 × 10^3^

**1** 309 2.7 × 10^3^ 464 0.172

360 1.7 × 10^3^ - -

419 1.6 × 10^3^ - -

^a^ Recorded in MeOH. ^b^ Excited at λ_S0→S1._^c^Determined by using quinine sulfate as standard(Φ_st_=0.54, 0.1 M H_2_SO_4_).

**Supplementary Table S4. Average Life time measurement of** 1

| **τ_1_** | **τ_2_** | **τ_3_** | **α_1_** | **α_2_** | **α_3_** | **χ^2^** | **<τ>** |
| --- | --- | --- | --- | --- | --- | --- | --- |
| 0.95 ns | 4.77 ns | 0.17 ns | 0.52 | 0.09 | 0.39 | 1.16 | 0.99 ns |

**Supplementary Table S5**. Comparison with other available fluorescent chemosensors

| **Serial No.** | **Type** | **LOD** | **Dynamic range** | **References** |
| --- | --- | --- | --- | --- |
| **1** | Rhodamine B-derivative | 1.8×10^-7^ M | 0-14 μM | *Sensors and Actuators B.* **211**, 325–331 (2015). |
| **2** | Triphenylvinyl derivative | 21.6 nM | 0-5 μM | *Anal.Chem*. **87**, 1470−147(2015) |
| **3** | Napthyridine-based rhodamine | 2.94×10^-5^ M | 0-12 μM | *RSCAdv*.**4**, 23428–23432 (2014). |
| **4** | Coumarin based | 1.0×10^-7^ M | 0-2 μM | *Inorg.Chem*.**49**, 7229–7231(2010). |
| **5** | Napthelene–pyrazol conjugate, | 31.78 nM | 0-1200 nM | *Analyst*. **139**, 4828–4835 (2014). |
| **6** | Furan derivative | 6.03×10^-7^ M | 0-8 μM | *Analyst*.**137**, 3975–3981 (2012). |
| **7** | Schiff base | 4.79× 10^-8^ M | 0-50 μM | *Sensors and Actuators B*. **216**, 86–104 (2015). |
| **8** | Naphthalene derivate | 1.0×10^-7^ M | 0-45 μM | *Sensors and Actuators B*. **197**, 200–205 (2014). |
| **9** | Salen type schiff base | **86** nM | 0-12 μM | **Present work** |

**Experimental Details**

**Materials**

Commercially available materials and reagent grade solvents were used as received. The common reagents and solvents were procured from Merck and Solvents were dried and distilled following the standard literature procedures prior to their use. 2-Hydroxy-1-napthaldehyde and 2,4,6-trimethylbenzene-1,3-diaminewere purchased from Sigma Aldrich Chemical Co., USA and used as received without further purifications. MEM and DMEM media were purchased by Himedia and Invitrogen respectively. DAPI, Hoechst, DNase and RNase were procured from sigma aldrich.

**X-ray Crystallography:**

Data were collected at 293 K using graphite-monochromated Mo Kα (λ_α_ = 0.71073 Å). The strategy for the Data collection was evaluated, by using the CrysAlisPro CCD software. The data were collected by the standard phi-omega scan techniques and were scaled and reduced using CrysAlisPro RED software. The structures were solved by direct methods using SHELXS-97 and refined by full matrix least squares with SHELXL-97, refining on *F^2^*^2^. The positions of all the atoms were obtained by direct methods. All non-hydrogen atoms were refined anisotropically. The remaining hydrogen atoms were placed in geometrically constrained positions and refined with isotropic temperature factors, generally 1.2 x *U_eq_* of their parent atoms*.* All the H-bonding interactions, mean plane analyses, and molecular drawings were obtained using the program Mercury (ver 3.1) and Diamond (ver 3.1d). The crystal and refinement data and H-bonding are summarized in **Supplementary Table S1**, **S2**.

**Quantum yield calculation:**,

The fluorescence quantum yields (*Φ*_F_) of **1** was calculated [eqn (1)] by the steady-state comparative method using quinine sulfate as a standard (Φst= 0.54)^1^

**Φ_F_ = Φ_st_**× **S_u_/S_st_**× **A_st_/A_u_**× **n^2^_Du_/n^2^_Dst_ (1)**

where Φ_F_ is the emission quantum yield of the sample, Φ_st_ is the emission quantum yield of the standard, A_st_ and A_u_ represent the absorbance of the standard and the sample at the excitation wavelength, respectively, while S_st_ and S_u_ are the integrated emission band areas of the standard and the sample, respectively, and n_Dst_ and n_Du_ are the solvent refractive index of the standard and the sample, and u and st refer to the unknown and the standard, respectively.

**Average life time measurement:**

The amplitude weighted lifetime was calculated using the following equation:

**<τ> = α_1_τ_1_+α_2_τ_2_ (2)**

where <τ> is the average ﬂuorescence lifetime of **1**. **τ_1_** and **τ_2_** are the average lifetime of various ﬂuorescent forms of compound **1** and α_1_ and α_2_ are the normalized pre-exponential factors. To obtain the best ﬁtting in all the cases the χ^2^ was kept near to unity.

**Detection limit calculation:**

The limit of detection (LOD) was calculated to be 86 nM by using following equation^3^

LOD = 3.3(σ/S) (1)

where σ is standard error and S is slope of calibration curve.

**DNA binding study**

**ETBr displacement assay:**

DNA binding ability of complex **1** was performed with calf-thymus (CT-DNA) in Tris-Hcl buffer (10µΜ Tris-HCl, pH =7.4). The fluorescence of EtBr intercalated with CT-DNA was taken as the control. The changes in the fluorescence intensities of the EtBr bound DNA were measured at 605 nm following excitation at 520 nm, with a gradual increase in concentration of1 (0 to 200μM in steps of 10μM each). EtBr intercalates with DNA bases emits strong fluorescence in the presence of DNA. When a quencher binds with DNA, it reduces the available binding sites for EtBr; hence, the fluorescence intensity decreases. Increasing the concentrations of **1**resulted in a gradual reduction in florescence intensity, which clearly indicates that **1** is replacing EtBr molecules from their respective binding sites. The fluorescence quenching spectra are shown in supplementary Fig. S20(a)

The fluorescence quenching results were studied by the Stern–Volmer equation^4^

*F*_0_/*F* = *K*_sv_ [Q] + 1 (1)

Where *F*_0_ is the emission intensity in the absence of quencher, *F* is the emission intensity in the presence of quencher, *K*_sv_ is the linear stern-volmer quenching constant and [Q] is the concentration of quencher. In the Stern–Volmer plots [inset in supplementary Fig. S20(a)] of F0/F *versus*[Q], the K_sv_ value is given by the slope. The binding constant and number of binding sites are determined by the scatchard equation^4^, which is given as

Log[F_0_-F/F] = logKa + *n* log[Q] (2)

Where K_a_ is the binding constant and *n* is the number of binding sites. The number of binding sites and the binding constant can be determined by the slope and the intercept of the scatchard plot (log(F_0_F)/F *vs* .log[Q], Supplementary Fig. S20(b). Binding constant and no. of binding site are given in the below table.

| **Compound** | **K_sv_(M^-1^)** | **K_a_(M^-1^)** | ***n*** |
| --- | --- | --- | --- |
| **1** | 3.8×10^4^ | 2.7×10^4^ | 1.09 |

***In vitro* Photostability study:**

Breast cancer cell line MCF-7 was seeded in three confocal dishes. Different dishes were incubated with 50 µL Hoechst (1 mg/mL), 50 µL FITC (1 mg/mL) and 50 µL **1** (1 mg/mL) respectively. The cells were imaged under confocal laser scan microscope using no delay scan mode and videos were recorded up to 3600 scan for FITC and **1** and 1500 scans for Hoechst. Hoechst was excited at 405 nm while FITC and **1** were excited at 488 nm. The most intense signal of first scan was considered for measuring relative decay in intensity. Images at every 200 scans were taken from the video and provided in figure **S21 (A)**. The relative intensity decay spectra upto 1500 scans are shown in figure **S21 (B)**.

**Supplementary References**

1. Saini, A. K., Srivastava, M., Sharma, V., Mishra, V. & Mobin, S. M. A highly selective, sensitive and reversible fluorescence chemosensor for Zn^2+^ and its cell viability. *Dalton Trans.* **45,** 3927–3935 (2016).

2. Sheldrick, G. M. Program for Crystal Structure Solution and Refinement; University of Göettingen: Göettingen, Germany, 1997*. A* **64,** 112–122 (2008).

3. Sharma, V., Saini, A. K. & Mobin, S. M. Multicolour fluorescent carbon nanoparticle probes for live cell imaging and dual palladium and mercury sensors. *J. Mater. Chem. B* **4,** 2466–2476 (2016).

4. Sharma, V. *et al.* Fabrication of innovative ZnO nanoflowers showing drastic biological activity. *New J. Chem.* **40,** 2145–2155 (2016).
